# Head and Body/Tail Pancreatic Carcinomas Are Not the Same Tumors

**DOI:** 10.3390/cancers11040497

**Published:** 2019-04-08

**Authors:** David Jérémie Birnbaum, François Bertucci, Pascal Finetti, Daniel Birnbaum, Emilie Mamessier

**Affiliations:** 1 Laboratoire Oncologie Prédictive, Centre de Recherche en Cancérologie de Marseille, INSERM UMR1068, CNRS UMR725, Aix-Marseille Université, 13273 Marseille, France; bertuccif@ipc.unicancer.fr (F.B.); finettip@ipc.unicancer.fr (P.F.); daniel.birnbaum@inserm.fr (D.B.); emilie.mamessier@inserm.fr (E.M.); 2 Faculté de Médecine, Aix-Marseille Université, 13385 Marseille, France; 3 Département de Chirurgie Générale et Viscérale, AP-HM, 13015 Marseille, France; 4 Département d’Oncologie Médicale, Institut Paoli-Calmettes, 13009 Marseille, France

**Keywords:** pancreatic cancer, tumor location, expression profiling, prognosis, survival

## Abstract

The association between pancreatic ductal adenocarcinoma (PDAC) location (head vs. Body/Tail (B/T)) and clinical outcome remains controversial. We collected clinicopathological and gene expression data from 249 resected PDAC samples from public data sets, and we compared data between 208 head and 41 B/T samples. The 2-year overall survival (OS) was better for the head than for the B/T PDACs (44 vs. 27%, *p* = 0.043), especially when comparing tumors with similar TNM classification (T3/4N0M0: 67% vs. 17%, *p* = 0.002) or from the same molecular class (squamous subtype: 31% vs. 0%, *p* < 0.0001). Bailey’s molecular subtypes were differentially distributed within the two groups, with the immunogenic subtype being underrepresented in the “B/T” group (*p* = 0.005). Uni- and multivariate analyses indicated that PDAC anatomic location was an independent prognostic factor. Finally, the supervised analysis identified 334 genes differentially expressed. Genes upregulated in the “head” group suggested lymphocyte activation and pancreas exocrine functions. Genes upregulated in the “B/T” group were related to keratinocyte differentiation, in line with the enrichment for squamous phenotype. We identified a robust gene expression signature (GES) associated with B/T PDAC location, suggesting that head and B/T PDAC are different. This GES could serve as an indicator for differential therapeutic management based on PDAC location.

## 1. Introduction

Pancreatic ductal adenocarcinoma (PDAC) is a major public health problem worldwide with 260,000 deaths annually [1], and its incidence is rising [2]. PDAC has the highest mortality rate of all human cancers [3]. Complete surgical removal of the tumor, followed by adjuvant chemotherapy, is the only curative treatment. However, less than 20% of patients are eligible for surgery [4]. The inoperability and the poor prognosis are due to late diagnosis rapid tumor progression (>50% of patients display metastases at diagnosis) [5], early recurrences after resection [6,7], and resistance to systemic therapies. Despite considerable research efforts during the past 20 years, conventional treatment approaches, including surgery, radiation, chemotherapy, and a combination of these, have achieved limited impact. Even after such treatments, most of the patients relapse and succumb from their disease. Molecular studies revealed that this poor benefit might be explained, at least in part, by the high heterogeneity found in pancreatic tumors.

Many studies have demonstrated that cancers of the right and left colons have different molecular characteristics [8,9,10,11,12,13,14], suggesting that carcinogenesis in a tissue may differ with tumor location. The clinical and biological symptoms are also often related to the location of the tumor. Because the pancreas is also subdivided in multiple anatomic regions—the uncinate process, the head, body, and tail—a long-lasting debate has been initiated to know whether PDAC location could have an impact on the developing tumor [15,16]. The fact that cell composition (Langerhans islets), fatty tissue infiltration, innervation, blood supply, and the frequency of the different types of pancreatic tumor (branch duct intrapapillary mucinous carcinoma tumor, mucinous cystic neoplasms) are slightly different between the head and the body/tail (B/T) zones of the pancreas legitimates the question. Studies focusing on the association between PDAC location (head vs. B/T) and clinical outcome (overall survival and disease-free survival) are extremely controversial [17,18,19]. Some of them reported that, at diagnosis, according to tumor stage, the survival of PDAC located in the B/T is better than the postoperative survival of PDAC located in the head [20,21]. In contrast, other studies showed that survival is similar between B/T and head tumor locations [16,22,23,24,25,26]. Finally, the SEER data (Surveillance, Epidemiology and End Results Program by the National Cancer Institute) [27], which has gathered the highest number of PDACs to date (1973–2002), showed that patients with a tumor located in the B/T have a worse survival than those with tumor located in the head (3-year survival: 3.9% vs. 6.2%) [20], and have a higher proportion of distant stage diseases (72.7% in B/T PDAC vs. 39.2% in head PDAC) [20]. This was recently confirmed in the Australian Pancreatic Cancer Genome Initiative (APGI) cohort (OS in B/T: 12.1 months versus Head: 22.0 months) [28].

Through in-depth molecular characterization, large-scale genomics provides the opportunity to address such a “long-lasting question”. Here, to determine whether head and B/T PDACs are similar or different diseases, we have studied clinicopathological and gene expression data of 249 resected pancreatic carcinoma samples, including 208 head and 41 B/T tumors.

## 2. Materials and Methods

### 2.1. Gene Expression Data Sets

We collected clinicopathological and gene expression data of 264 clinical pancreatic carcinoma samples from four public data sets, including 249 operated primary cancer samples with informed cancer localization (Supplementary Table S1).

### 2.2. Preanalytic Gene Expression Data Processing

Before analysis, gene expression data were processed. First, each data set was normalized separately: quantile normalization for the available processed data from Illumina set and Robust Multichip Average (RMA) [29] with the nonparametric quantile algorithm as normalization parameter for the raw Affymetrix data. Normalization was done in R using Bioconductor and associated packages. Second, we mapped hybridization probes across the technological microarray platforms represented in these data sets. We used NetAffx Annotation files to update the Affymetrix gene chips annotations, and both SOURCE and EntrezGene (Homo sapiens gene information db, release from 9 December 2008) to retrieve and update the non-Affymetrix gene chips annotations. Then, all probes were mapped based on their EntrezGeneID. In the case of multiple probes mapped to the same EntrezGeneID, the one with the highest variance in a particular dataset was selected to represent the EntrezGeneID. For the TCGA and Bailey’s data, we used the available normalized RNA-Seq data that we log2-transformed. Samples of the four sets were pooled for unsupervised analysis by using COMBAT (empirical Bayes) as batch effect removal method, included in the inSilicoMerging R/Bioconductor package. The merged set included 14,531 genes in log2-transformed data. The accuracy of normalization was verified by principal component analysis (PCA) (Supplementary Figure S1). Hierarchical clustering was done using Cluster program and displayed using Treeview [30].

### 2.3. Gene Expression Data Analysis

We first defined the molecular subtypes of pancreatic cancer samples in each data set separately as defined in the original publications: the three Collisson’s subtypes [31] were classical, quasi-mesenchymal, and exocrine-like; the two Moffitt’s epithelial subtypes [32] were basal-like and classical; the two Moffitt’s stromal subtypes were normal and activated; and the four Bailey’s subtypes [33] were squamous, pancreatic progenitor, immunogenic, and ADEX. Then, we applied the 20 Gatza’s activation pathway signature [34] and the 64 immune and stroma cell types classifiers using the xCell web tool [35]. Finally, to identify a gene expression signature able to distinguish PDAC of the head from PDAC of the body/tail of the pancreas, we applied a supervised analysis using learning and validation sets. The learning set included 137 samples of the TCGA data set, including samples from 116 patients in the “head group” and samples from 21 patients in the “B/T group”. To identify the differentially-expressed genes, a moderated *t*-test analysis was applied to expression levels of each gene using linear models with empirical Bayes statistic included in the limma R package [36]. Genes were considered as significantly differentially expressed if they showed an absolute fold change ≥2, a *p*-value ≤ 5%, and a *q*-value ≤ 25%. The classification model was then computed using a metagene-based approach with the significant genes and statistics for ponderation. Using a cut-off of 0, samples were classified as “head-like” or “body/tail-like”. Robustness of the resulting classification model was assessed in the independent validation set (*n* = 112 samples, including 92 head samples, and 20 B/T samples), i.e., the three remaining sets Analyses were done in R and associated packages. Ontology analyses were performed using DAVID Bioinformatics Resources 6.8 [37].

### 2.4. Statistical Analysis

Overall survival (OS) was calculated from the date of diagnosis to the date of death from pancreatic cancer. Follow-up was measured from the date of diagnosis to the date of last news for living patients. Survivals were calculated using the Kaplan–Meier method and were compared with the log-rank test. Uni- and multivariate prognostic analyses were done using Cox regression analysis (Wald test). All statistical tests were two-sided at the 5% level of significance. We followed the reporting REcommendations for tumor MARKer prognostic studies (REMARK criteria) [38].

## 3. Results

### 3.1. Patients’ Characteristics

A total of 249 PDAC samples were included in the analysis. Their clinicopathological and molecular data are summarized in Table 1. Briefly, the median age was 66 years (range 32–90) and 117 patients (47%) were female. Most tumors were of ductal type (*n* = 219, 88%) and were classified as AJCC stage II tumors (*n* = 209, 84%). Following the TNM classification, most samples were pT3 tumors (*n* = 148; 79%), most had at least one lymph node involved *(n* = 162; 67%), but only 10 had detectable metastases at diagnosis (4%). Within the whole population, 26 tumors (10%) were located in the tail, 15 (6%) in the body, and 208 (84%) in the head of the pancreas. The 2-year OS of the whole population was 41% (95% CI: 0.33–0.49), with a median follow-up of 11 months (range: 0–156.4).

Samples were then characterized at the molecular level. The Bailey’s classification identified the four subtypes: ADEX (21%), immunogenic (18%), pancreatic progenitor (22%), and squamous (39%). Regarding the Colisson’s classification, 47%, 35%, and 18% of PDACs were classified as classical, exocrine-like, and quasi-mesenchymal, respectively. According to the Moffitt’s “Tumor” classification, 45% of samples were basal-like and 5% were classical. The Moffitt’s “Stroma” classification was distributed as activated (63%) and normal (37%).

### 3.2. Clinicopathological and Molecular Characteristics According to Anatomic Location

Patients were divided in two groups based on the tumor anatomic location: “head” or “B/T” of the pancreas. A total of 208 patients (84%) had a “head” PDAC and 41 (16%) had a “B/T” PDAC. As shown in Table 1, patients from the “B/T group” were older (*p* = 0.039) than patients from the “head group”, had more pT1 tumors (*p* = 0.049), less pathological lymph node involvement (*p* = 0.001), less tumors from ductal type (*p* = 0.015), and less stage 2 tumors (*p* = 0.04). No difference between the two groups was observed concerning the tumor grade.

In molecular term, all Bailey’s molecular subtypes were represented in both “head” and “B/T” tumors. However, the distribution was significantly different, notably the immunogenic subtype was almost absent from the “B/T group” (*p* = 0.0058). Accordingly, the immunogenic subtype was more frequent in the “head group” than in the “B/T group” (*p* = 0.0058). No significant difference was observed between the two location groups regarding the Collison’s subtypes and the Moffit’s tumor subtypes. By contrast, there was more Moffit’s activated stroma subtype in the “B/T group” than in the “head group” (*p* = 0.006).

### 3.3. OS According to Anatomic Location and Prognostic Analysis

**Figure 1:**
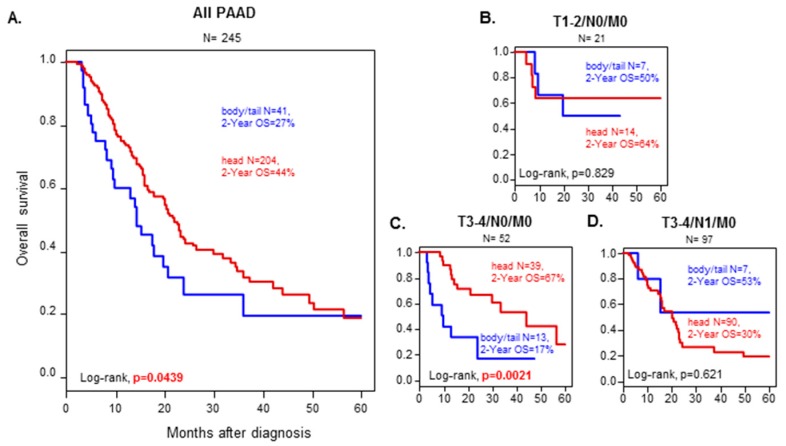
Overall survival in Head or Body/Tail tumors. Kaplan–Meier overall survival (OS) curves according to PDAC location (**A**). All pancreatic ductal adenocarcinoma (PDAC) and (**B**) T1/2 N0 M0 tumors. (**C**) T3/4N0M0 tumors. (**D**) T3/4 N1 M0 tumors. T1/2N1M0 tumors have less than 10 samples in total and were not graphed). Head samples are in yellow. Body/Tail samples are in blue. The *p*-values (log-rank test) for the comparison between the two classes within each molecular subtype are indicated.

Median OS was 21.9 months (range: 1–156) in the “head group” and 14.1 months (range: 1–60) in the “B/T group”, and the respective 2-year OS were 44% (95% CI: 0.36–0.54) with a median follow-up of 11 months, versus 27% (95% CI: 0.14–0.49) with a median follow-up of 9.6 months, revealing longer survival in the “head group” than in the “B/T group” (*p* = 0.0439; Figure 1A).

We then compared the prognostic value of the head versus B/T anatomic location with that of other clinicopathological variables and molecular subtype classifiers (Bailey, Collisson, and Moffitt). In univariate analysis (Table 2), the pN status (*p* = 0.0078), the PDAC location (*p* = 0.0453), the Bailey (*p* = 0.006), and Moffitt (“Tumor” *p* = 0.00076 and “Stroma” *p* = 0.0002) classifiers were associated with OS (Wald test). The hazard ratio (HR) for death was 0.63 (95% CI: 0.41–0.99) in the head vs. B/T classes. In multivariate analysis, the PDAC location (*p* = 0.0047), the pN status (*p* = 0.0028), Moffitt tumor (*p* = 0.0497), and stroma classifiers (*p* = 0.0232) remained significant, suggesting independent prognostic value.

Since AJCC TNM staging is the major prognostic factor used in clinical practice, we analyzed OS in comparable situations, i.e., in groups matched according to the TNM staging. The “head group” conserved a better 2-year OS (67%) than the “B/T group” (17%) when looking at tumors classified as T3-4N0M0 (*p* = 0.002; Figure 1B–D).

**Figure 2:**
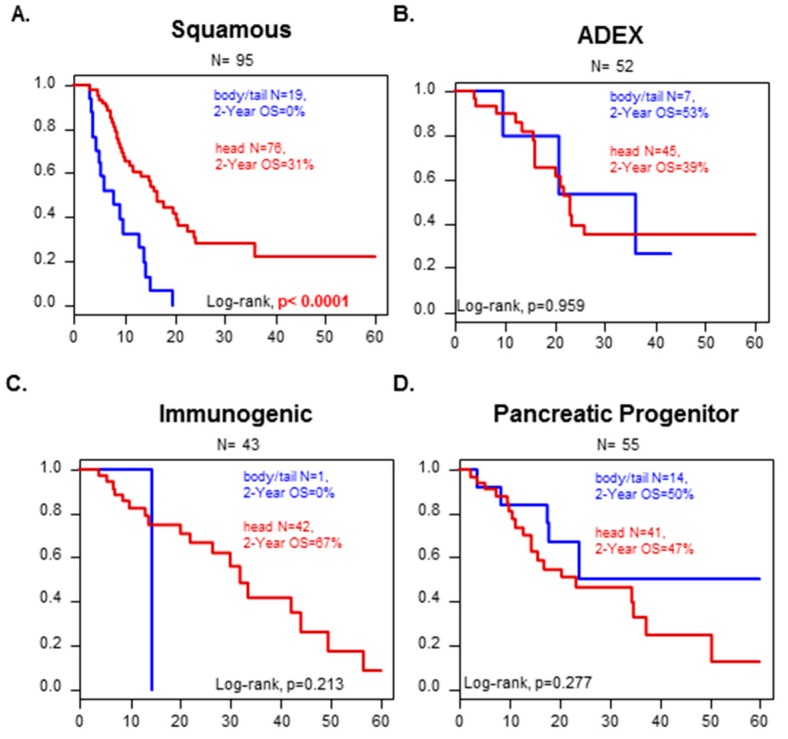
OS in the validation set according to Head or Body/Tail in the different Bailey’s molecular subtypes. Kaplan–Meier OS curves according to PDAC location (Head vs. B/T) and the molecular subtypes defined by Bailey (**A**) Squamous, (**B**) ADEX, and (**C**) Immunogenic, and (**D**) Pancreatic progenitor. The *p*-values (log-rank test) for the comparison between the two classes within each molecular subtype are indicated.

We looked at how the molecular classification could affect the survival in each of the two location groups. Notably, tumors from the “squamous” subtype from the Bailey’s classification had a worse overall survival when the tumor was from the “B/T group” than from the “head group” (2-years OS: 0% versus 31%, *p* < 0.0001) (Figure 2). It was not possible to conclude for the immunological subtype because “B/T” tumors included only one case. No difference in OS was observed with the other molecular subtypes according to the PDAC location.

### 3.4. Head versus Body/Tail Gene Expression Signature

**Figure 3:**
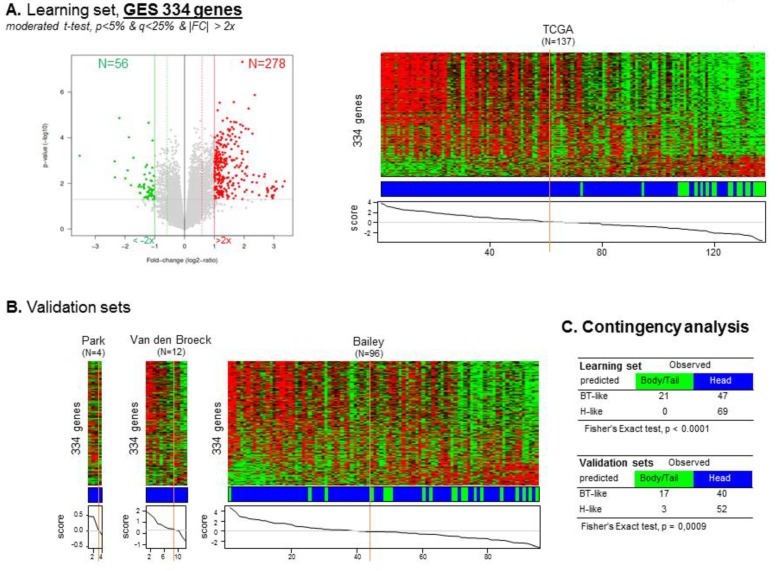
Establishment of the 334-gene expression signature between Head and B/T tumors based on the learning and validation sets. (**A**) Volcano plot identifying 334 genes differentially expressed (GES) between Head and B/T tumors. This volcano plot was obtained using a moderated *t*-test, *p* < 5% & *q* < 25%, |FC| > 2× between the Head and B/T tumors (left). The GES was used to classify the samples from the TCGA learning set (right). (**B**) Classification of the samples from each of the three validation sets using the 334 GES. (**C**) Contingency analyses of the classification in the learning and validation sets using the 334 GES identified from the learning set.

To identify a gene expression signature (GES) of “head” versus “B/T” PDAC, we used the TCGA cohort as a learning set (*n* = 137) and the other sets pooled as a validation set (*n* = 112). A total of 334 genes were differentially expressed, including 278 (83%) genes upregulated and 56 (17%) genes downregulated in the “head group” compared to the “B/T group” (Supplementary Tables S2 and S3). A “location” classifier was then built from this gene list, and as expected, accurately classified the B/T samples according to their actual location (Fisher’s exact test, *p* < 0.0001). Importantly, its robustness as a classifier for B/T samples was confirmed in the independent validation set (*p* = 0.0009, Fisher’s exact test) (Figure 3).

Regarding the 278 genes identified as upregulated in the “head group” (Supplementary Tables S2 and S3), two major pathways were noticeable. The first one was related to immune response activation (*p* < 0.0001), more specifically involving genes from inflammatory response (*p* < 0.0001), adaptive immune response (*p* < 0.0001), G-protein-coupled receptor signaling pathway (*p* < 0.0001), transmembrane receptor protein tyrosine kinase signaling (*p* < 0.0001), cell surface receptor signaling (*p* < 0.0001), and cellular defense response (*p* < 0.0001) pathways. Such activation of “Immune-related” pathways is coherent with above-mentioned enrichment of the immunogenic subtype in the “head group”. The immune signature showed a strong immune infiltrate, more specifically involving upregulation of mRNA related to unconventional γδ-T cells (*p* < 0.0001), to the presence of a cytotoxic activity potentially at work and B-cells-related markers (*p* < 0.003). Finally, numerous cytokines, chemokines, and related receptors were secreted in “head” tumors and involved in immune cell recruitment and function (*p* < 0.0001).

The second major pathway upregulated in the “head” PDAC was related to pancreas function, notably triglyceride catabolic process (*p* < 0.0001), proteolysis (*p* < 0.0001), and digestion (*p* < 0.0001) process. These genes were related to pancreatic secretion (*p* < 0.0001), fat, protein and vitamin digestion and absorption (*p* < 0.0001), and glycolysis/gluconeogenesis/drug metabolism (*p* < 0.01).

Regarding the 56 genes identified as downregulated in the “head” compared to “B/T” PDAC (Supplementary Tables S2 and S3), associated pathways were involved in epidermis development and keratinocyte differentiation (*p* < 0.0001), more specifically to squamous tumors differentiation. Some of the downregulated genes were also related to the nervous system, notably dopaminergic synaptic transmission (*p* < 0.0001) and positive regulation of calcium ion import (*p* < 0.0001) that function as regulators of pancreas endocrine function, such as glucagon synthesis and positive regulation of cAMP biosynthetic process (*p* < 0.0001). Finally, several genes were related to tumor aggressiveness in “B/T” tumors, including genes involved in cellular proliferation, survival, and epithelial to mesenchymal transition. Some of these genes are associated components of the EGFR and/or ERBB and/or TP53 pathways. Other genes are involved in the regulation of cell proliferation and local invasion and the acquisition of drug resistance mechanisms and/or TGF-β-induced EMT.

### 3.5. Correlation between “Head” vs. “Body/Tail” Locations and Immune and Stromal Signatures

Because tissues are complex mixtures consisting of numerous noncancerous cell types, we searched for correlation between “head” vs. “body/tail” transcriptomic profiles and a variety of innate and adaptive immune cells, stromal cells, and many other cell types that are found in the tumor. For this, we used the *XCELL* [35] and the Gatza [39] gene expression signatures. In brief, the Gatza gene signature-based data aims at identifying enrichment for distinct oncogenic pathway activities; the *XCELL* gene signature-based data allow identifying immune and stromal cell types present within the tumor.

Those signatures showed correlations between the “head” transcriptional PDAC profile and the T and B immune infiltrates (CD8pos_Tcm, CD4pos_T.cells, naive_B.cells, B.cells, and Memory_B.cells) as well as some antigen-presenting cells (pDC, aDC, and B cells). Accordingly, the Microenvironment_Score (*p* = 0.0005) and Immune_Score (*p* = 0.0006) were significantly correlated with the “head” PDAC (Supplementary Table S4). By contrast, the “B/T” PDAC transcriptional profile was correlated with Keratinocytes (*p* = 0.0007), Epithelial_cells (*p* = 0.0017), Smooth_muscle (*p* = 0.0123), and TGFβ (*p* = 0.0299) gene signature-based profiles. As mentioned earlier, these signatures are suggestive of ongoing epithelial-to-mesenchymal transition (EMT) process. Altogether, these results confirmed the results of our “location” GES in term of differential pathways involved in the “head” versus “B/T” PDAC.

## 4. Discussion

Currently, the AJCC TNM classification is the only prognostic factor used in clinical practice to assess survival of resected PDAC and guide treatments decision. However, recent data suggest that PDAC localized in the “head” might have a better survival than PDAC localized in the “B/T” zone of the pancreas [20,27]. So far, this information is not taken into account when using the TNM classification. Still, the association between PDAC location and survival has been the subject of a longstanding debate, which is not completely settled yet. Here, we reported longer 2-year OS in “head” PDAC than in “B/T” PDAC. It has been hypothesized that “head” PDAC comes to clinical attention earlier than “B/T” PDC due to early detectable symptoms, such as high carbohydrate antigen 19-9 (CA19-9) positivity or painless jaundice (caused by tumor obstruction of the bile ducts, which pass through the head of the pancreas). Thus, clinicians are prompt to perform abdominal imaging that will reveal the underlying tumor, and may thus deal with “earlier” stages of PDAC tumors. In contrast, tumors of the body and tail do not produce jaundice, and therefore most often come to clinical attention later, once weight loss and/or abdominal pain become apparent [20,27,40]. However, reciprocally, patients primarily diagnosed with pancreatic “B/T” cancer are associated with much more pain, higher serum albumin level, higher carcinoembryonic antigen (CEA), and higher metastasis rate. Whatever the cause of detection, at diagnosis, “B/T” PDAC corresponds to larger tumors that bear numerous molecular alterations, suggesting a later stage of disease evolution [20,41,42,43,44,45,46,47,48]. They are also more heterogeneous at the genomic level [49]. To avoid bias that can affect survival, we compared tumors with the same histological TNM classification. We confirmed that “B/T” PDAC has the worst 2-year OS for T3 and T4 N0 M0 tumors. This was not significant in the other groups, most certainly due to an inadequate number of samples in the analyzed groups despite our large cohort. In multivariate analysis, the pN status, the PDAC location and the Moffitt subtypes were the only three significant factors. The stratified hazard ratio (HR) for death was 0.46 (95% CI 0.27–0.79) in the head versus B/T classes. We thus confirmed than PDAC localization is an independent prognostic factor when compared with the molecular subtype classifications. Of note, the location remained an independent prognostic factor in multivariate analysis when analysis was limited to the ductal tumors only (data not shown). We cannot exclude that this difference in survival might be inherent to other characteristics. We may however rule out the impact of the surgical procedure associated to the location (distal pancreatectomy versus pancreaticoduodenectomy). Indeed, when considering the survival of PDAC according to the molecular subtype classification (Figure 2B–D, *n* = 150 cases), there was no significant difference in survival between the Head and the B/T groups except for the Squamous subtype. If location and associated surgery only had an impact on survival, it would have been detectable for all molecular subtypes of PDAC, and not the Squamous subtype only. It is also possible that the molecular differences related to the head versus B/T locations are not tackled by the molecular subtypes.

Overall, we were interested in defining the differential gene expression signature related to PDAC location. A recent study used pre-established “genes programs” issued from the PDAC classification of Bailey [33] to conduct a similar analysis [28]. It showed that the location in the “B/T” was associated with the squamous subtype of PDAC, the molecular subtype with the worse clinical outcome. In addition to this finding, we reported a worst survival for squamous tumors localized in the B/T as compared to the ones isolated from the “head” of the pancreas. This means that, independently from the squamous classification, the PDAC location has a prognostic impact. To go further with minimal induced bias (which can be introduced using pre-established gene programs), we did a global supervised analysis on a large learning dataset, then validated the obtained GES on an independent set. By doing so, we qualitatively identified the transcriptional difference between the two PDAC anatomic locations.

In line with the shorter survival of B/T tumors, our GES analysis between “head” and “B/T” PDAC revealed that B/T tumors were probably more aggressive tumors, being highly proliferative and more prone to EMT. The higher proliferation rate could explain why “B/T” tumors often display bigger size at diagnosis [28]. Tumor recurrence might also be related to this EMT-prone status [28]. Interestingly, these effects (proliferation and EMT status) seem to be amenable to EGFR and or ERBB2 regulation. *TNS4, NTSR1*, and *PNCK* are three genes involved in these pathways. Tensin-4 (or TNS4) functions as an oncogene and promotes cell proliferation and/or motility in numerous solid tumors, including pancreatic cancer [50], gastric cancer, hepatocellular carcinoma, and colorectal cancers. TNS4 is upregulated by EGF-induced ERK1/2 activity and KRAS, and as a retro-control loop, TNS4 regulates EGFR expression. Similarly, NTSR1, which belongs to the large superfamily of G-protein coupled receptors, was shown to promote tumor invasion by accelerating EMT [50]. NTSR1 could also affect cancer cells proliferation. High NTSR1 expression is associated with pancreatic ductal adenocarcinoma [51] and is a poor prognosis factor. Finally, PNCK, a member of the calcium/calmodulin-dependent protein kinase family of protein serine/threonine kinases, was shown to induce ligand-independent EGFR degradation. High expression of PNCK resulted in increased proliferation, clonal growth, cell cycle progression, and trastuzumab resistance in ERBB2-positive tumor cells [52,53,54]. Altogether, those three genes deserve more attention and might represent attractive therapeutic targets for “B/T” PDAC.

Because Langerhans islet concentration is much higher in the tail than in the head of the pancreas, theoretically, patients with pancreatic “B/T” cancer are more prone to have islet dysfunction and subsequent diabetes. Data comparing the onset of pancreatic cancer-induced diabetes between “head” and “B/T” PDAC are limited. Here, we have shown that the endocrine functions of the pancreas seem to be relatively more efficient in the “B/T” than in the “head” PDAC. Even in the presence of the growing tumor, the endocrine cells, notably insulin-secreting cells, seem to maintain their function. Insulin-positive endocrine cells seem to be “refractory” to malignant progression. This can explain why postoperative diabetes is higher in patients receiving distal (B/T) pancreatectomy than in those treated with pancreaticoduodenectomy [8,55] and the delayed diagnosis of “B/T” tumors in the absence of any detectable symptoms.

Another striking information related to our analysis was the absence of the immunological subtype in “B/T” PDAC. This highlights a profound defect in the recruitment of leukocytes within “B/T” PDAC and suggests that these tumors are probably noneligible for immune therapy-based treatment. The obvious defect in immune response was mainly noticeable due to the absence of CD8 γδ−T cells and B cells in “B/T” PDAC. Because this transcriptomic analysis results from the whole tumor piece, we cannot know whether these cells were infiltrating or surrounding the tumor. These cells however overexpressed immune checkpoint inhibitory molecules, such as *PD1*, *BTLA*, *TNFSF14 (LIGHT)*, and *TIGIT*, which are the sign of immune exhaustion or inhibition of tumor infiltrating lymphocytes. Gamma-delta T cells are part of the unconventional T cells involved in the initiation and propagation of immune responses. It is thus plausible that the reactivation of this cytotoxic immune infiltrate, achievable with the help of immune checkpoint inhibitors and/or potent analogues with more or less selective stimulatory activity for γδ-T cells (such as bromohydrin diphosphate (BrHPP), anti-KIR, phosphoantigen, or ligation of NKG2D, for example), can benefit to “head” PDAC. As for many solid tumors, boosting the immune system, especially with neoadjuvant therapeutic agents might be highly beneficial for “head” PDAC.

## 5. Conclusions

**Figure 4:**
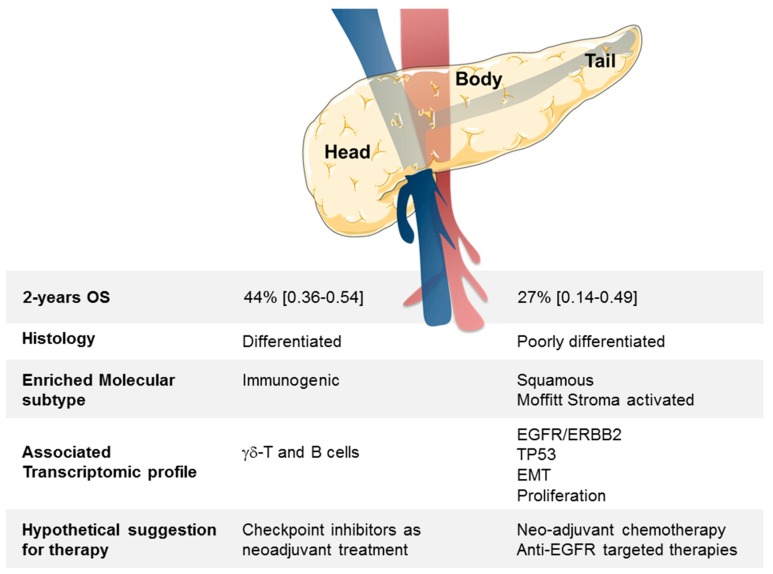
Summary of clinical, histologic, and molecular differences between head and B/T PDAC tumors.

In conclusion, we have identified a robust GES associated with B/T PDAC samples. The nature of genes differentially expressed suggests that different therapeutic managements might be applied in clinics depending on the “head” or the “B/T” location of tumor (Figure 4).

## Figures and Tables

**Table 1:** Clinicopathological and molecular characteristics of patients in the whole population and in each location group.

Characteristics	N	All	Pancreas Cancer Site		*p*-Value	Statistic
			Body/Tail	Head		
Age at diagnosis, years					0.198	0.56
≤60	79	79 (32%)	9 (22%)	70 (34%)		(0.22–1.27)
>60	170	170 (68%)	32 (78%)	138 (66%)		
Sex					0.733	0.86
Female	117	117 (47%)	18 (44%)	99 (48%)		(0.41–1.78)
Male	132	132 (53%)	23 (56%)	109 (52%)		
Pathological tumor size (pT)					0.049	
pT1	10	10 (5%)	5 (16%)	5 (3%)		
pT2	24	24 (13%)	4 (13%)	20 (13%)		
pT3	148	148 (79%)	22 (71%)	126 (81%)		
pT4	5	5 (3%)	0 (0%)	5 (3%)		
Pathological lymph node status (pN)					0.001	3.3
Negative	79	79 (33%)	22 (56%)	57 (28%)		(1.53–7.1)
Positive	162	162 (67%)	17 (44%)	145 (72%)		
Metastases					0.063	0.28
Negative	239	239 (96%)	37 (90%)	202 (97%)		(0.06–1.4)
Positive	10	10 (4%)	4 (10%)	6 (3%)		
AJCC stage					0.048	
1	25	25 (10%)	7 (17%)	18 (9%)		
2	209	209 (84%)	30 (73%)	179 (86%)		
3	5	5 (2%)	0 (0%)	5 (2%)		
4	10	10 (4%)	4 (10%)	6 (3%)		
Pathological grade					0.808	
1	21	21 (9%)	2 (5%)	19 (9%)		
2	137	137 (57%)	23 (57%)	114 (57%)		
3	81	81 (34%)	15 (38%)	66 (33%)		
4	2	2 (1%)	0 (0%)	2 (1%)		
Bailey subtypes					0.006	
ADEX	52	52 (21%)	7 (17%)	45 (22%)		
Immunogenic	45	45 (18%)	1 (2%)	44 (21%)		
Pancreatic progenitor	56	56 (22%)	14 (34%)	42 (20%)		
Squamous	96	96 (39%)	19 (46%)	77 (37%)		
Collisson subtypes					0.065	
Classical	118	118 (47%)	24 (59%)	94 (45%)		
Exocrine-like	87	87 (35%)	8 (20%)	79 (38%)		
Quasi-mesenchymal	44	44 (18%)	9 (22%)	35 (17%)		
Moffitt subtypes, “Tumor”					0.395	1.4
Basal-like	112	112 (45%)	21 (51%)	91 (44%)		(0.65–2.8)
Classical	137	137 (55%)	20 (49%)	117 (56%)		
Moffitt subtypes, “Stroma”					0.007	3.1
Activated	154	154 (63%)	32 (82%)	122 (60%)		(1.26–8.72)
Normal	90	90 (37%)	7 (18%)	83 (40%)		
Follow-up median, months (min-max)	245	11.17 (0–156.4)	9.63 (0.03–60.25)	10.97 (0–156.4)		
2-Year OS (95% CI)	245	41% (0.33–0.49)	27% (0.14–0.49)	44% (0.36–0.54)	0.044	

ADEX aberrantly differentiated endocrine exocrine; AJCC American Joint Committee on Cancer; CI confidence interval; OS overall survival.

**Table 2:** Uni- and multivariate Cox regression analyses for overall survival (validation set).

Characteristics		Univariate			Multivariate		
		N	HR (95%CI)	*p*-Value	N	HR (95%CI)	*p*-Value
Age at diagnosis	>60 vs. ≤60	245	0.93 (0.63–1.37)	0.708			
Sex	male vs. female	245	1.13 (0.78–1.62)	0.516			
Pathological type	other vs. ductal	245	1.12 (0.62–1.99)	0.711			
Pathological tumor size (pT)	2 vs. 1	183	1.78 (0.37–8.66)	0.290			
	3 vs. 1		2.49 (0.61–10.24)				
	4 vs. 1		5.88 (0.81–42.62)				
Pathological lymph node status (pN)	1 vs. 0	237	1.77 (1.16–2.7)	**0.008**	230	2.04 (1.28–3.27)	**0.003**
Metastases	1 vs. 0	245	1.71 (0.74–3.92)	0.207			
AJCC stage	2 vs. 1	245	2.14 (0.99–4.63)	0.083			
	3 vs. 1		4.98 (1.01–24.50)				
	4 vs. 1		3.50 (1.16–10.61)				
Pathological grade	2 vs. 1	241	1.47 (0.59–3.69)	0.056	230	1.38 (0.52–3.61)	0.516
	3 vs. 1		2.33 (0.92–5.89)		230	1.86 (0.71–4.89)	0.209
	4 vs. 1		2.52 (0.49–13.13)		230	3.61 (0.66–19.82)	0.140
Pancreatic tumor site	head vs. body/tail	245	0.63 (0.41–0.99)	**0.045**	230	0.46 (0.27–0.79)	**0.005**
KRAS mutation	MT vs. WT	137	1.2 (0.68–2.13)	0.534			
KRAS mutation, exon12	G12D vs. WT	137	1.53 (0.79–2.98)	0.317			
	G12R vs. WT		1.03 (0.39–2.72)				
	G12V vs. WT		0.63 (0.26–1.57)				
Bailey subtypes	Immunogenic vs. ADEX	245	0.92 (0.50–1.70)	**0.006**	230	1.64 (0.75–3.6)	0.216
	Pancreatic progenitor vs. ADEX		0.97 (0.54–1.73)		230	1.43 (0.68–3)	0.345
	Squamous vs. ADEX		1.87 (1.13–3.10)		230	1.81 (0.8–4.1)	0.152
Collisson subtypes	Exocrine-like vs. Classical	245	1.13 (0.74–1.70)	0.131	230	1.75 (0.98–3.12)	0.057
	Quasi-mesenchymal vs. Classical		1.64 (1.01–2.66)		230	1.12 (0.63–2.01)	0.698
Moffitt subtypes, “Tumor”	Classical vs. Basal-like	245	0.53 (0.37–0.77)	**0.001**	230	0.55 (0.31–1)	**0.050**
Moffitt subtypes, “Stroma”	Normal vs. Activated	240	0.46 (0.3–0.69)	**0.0002**	230	0.58 (0.36–0.93)	**0.023**

ADEX: aberrantly differentiated endocrine, CI confidence interval, HR hazard ratio; *p*-value in bold: statistically significant.

## Supplementary Materials

The following are available online at https://www.mdpi.com/2072-6694/11/4/497/s1, Table S1: List of pancreatic cancer data sets included. Table S2: List of 334 genes differentially expressed between head and B/T location of PDAC. Table S3: Ontology analysis of the 334 genes differentially expressed between the head and B/T location of PDAC. Table S4: Enrichment for cell types and or oncogenic pathway activities in “head” vs. “body/tail” samples. Figure S1. Principal component analysis performed before and after normalization of the 4 data sets.
