# Supplementary material for: Head and Body/Tail Pancreatic Carcinomas Are Not the Same Tumors
Source: Cancers (Basel). 2019 Apr 8;11(4):497. doi: 10.3390/cancers11040497 (PMC6520848; doi:10.3390/cancers11040497)
Supplement: Supplementary file 1 [file cancers-11-00497-s001.pdf]

cancers

MDPI

Article

Head and Body/Tail Pancreatic Carcinomas Are Not the Same Tumors

David Jérémie Birnbaum, François Bertucci, Pascal Finetti, Daniel Birnbaum and Emilie Mamessier

Supplementary Material

Table S1. List of pancreatic cancer data sets included.

Reference	Source of data	Technological platform	N° of probe sets/genes	TNM edition	All samples	Primary pancreatic samples included in the present analysis	Primary pancreatic samples with location included in the present analysis
Van den Broeck et al., J Exp Clin Cancer Res 2012	GEO database, GSE42952	Affymetrix, array U133 Plus 2.0	54K	7th (few cases were included before 2002)	23 (7%)	12 (5%)	12 (5%)
Park et al., Mod Pathol 2014	GEO database, GSE43795	Illumina, array Human HT-12 V4.0	48K	7th	31 (9%)	6 (2%)	4 (2%)
TCGA, PAAD	TCGA portal, https://tcga-data.nci.nih.gov	Illumina, RNA sequencing V2	25K	7th (few cases were included before 2002)	183 (55%)	150 (57%)	137 (55%)
Bailey et al., Nature 2016	European Genome-phenome Archive (EGA), EGAS00001000154	Illumina, RNA sequencing HiSeq	18K	6/7th	96 (29%)	96 (36%)	96 (38%)
			Sum		333	264	249

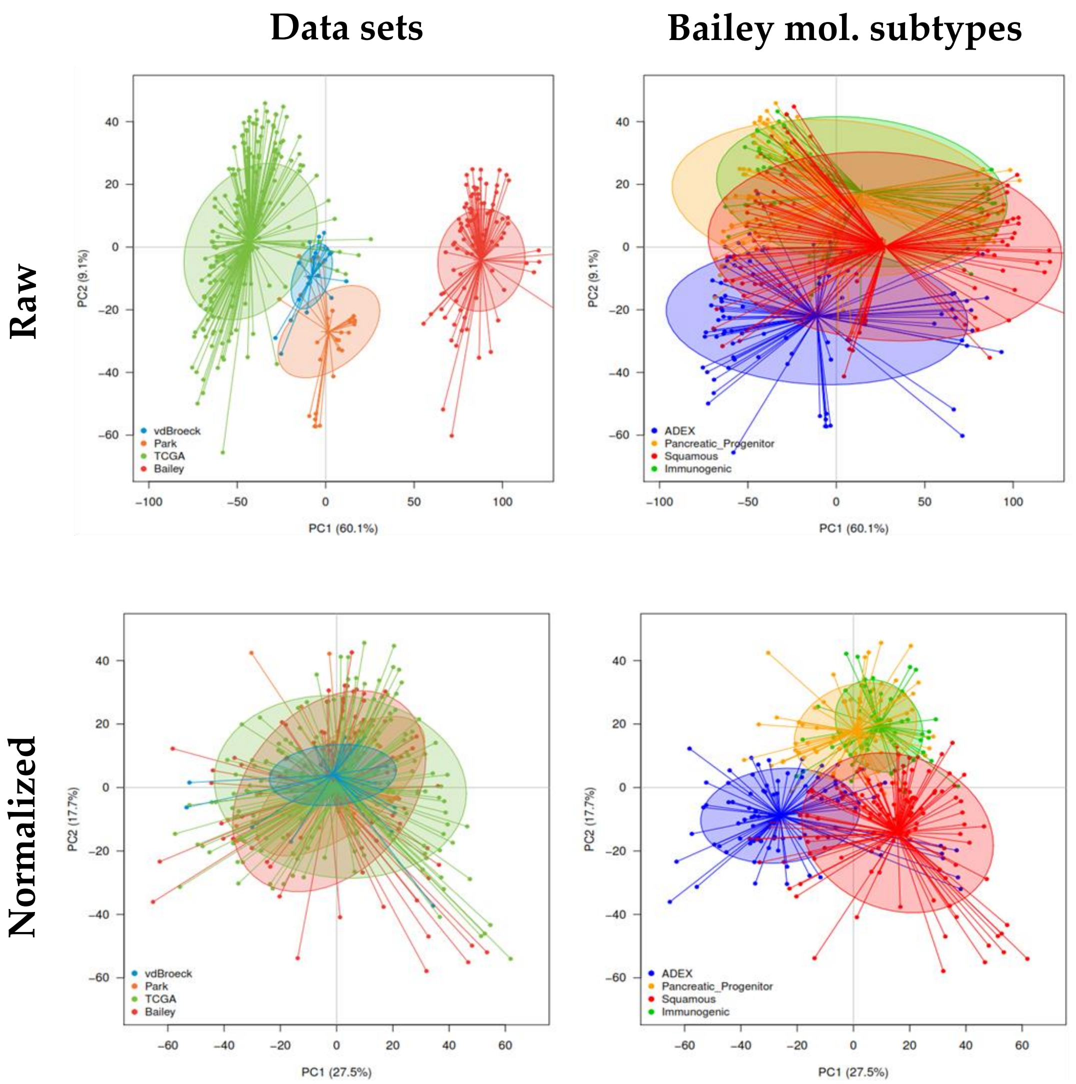

Figure S1. Principal component analysis performed before and after normalization of the four data sets. PCA was applied to the 249 resected PDAC samples and the 859 Bailey's subtype genes common to all data sets. A. Before normalization, the PDAC samples in the 2D scatter plot representation are grouped according to their origin data set (left: each color represents a set) and not according to the Bailey's subtypes (right: blue for ADEX, orange for pancreatic progenitor, green for immunogenic and red for squamous). B. After normalization, samples are correctly clustered according to their molecular subtypes (right), rather than to their origin data set (left), suggesting that the standardization has removed technical differences in gene expression while maintaining the information relevant to biological differences. Color code: Van den Broeck (Orange); Park (Blue); TCGA (Green); Bailey (Red).

Table S2. List of 334 genes differentially expressed between head and B/T location of PDAC. This GES was obtained from the Learning set (TCGA's data), composed of 116 head and 21 body/tail PAAD samples. The GES identified 334 differentially regulated genes using a moderated t-test, p<5%, q<25% and a Fold change (|FC|) > 2x.

PID	Gene.Symbol	Entrez Gene ID	Body/Tail (average, log2)	Head (average, log2)	Log2-ratio (H vs. B/T)	t	p-value	q-value	Associated to
GFRA1|2674	GFRA1	2674	5.76	7.70	1.94	5.78	4.72E-08	4.62E-04	Up head vs. body/tail
DARC|2532	DARC	2532	6.52	8.88	2.36	5.06	1.35E-06	6.58E-03	Up head vs. body/tail
SELP|6403	SELP	6403	6.07	7.73	1.65	4.89	2.75E-06	7.14E-03	Up head vs. body/tail
MPZ|4359	MPZ	4359	4.09	5.27	1.18	4.88	2.92E-06	7.14E-03	Up head vs. body/tail
FLT3|2322	FLT3	2322	3.17	5.33	2.15	4.74	5.23E-06	1.02E-02	Up head vs. body/tail
CCDC69|26112	CCDC69	26112	8.12	9.23	1.11	4.70	6.29E-06	1.03E-02	Up head vs. body/tail
GZMH|2999	GZMH	2999	3.80	5.23	1.43	4.51	1.39E-05	1.54E-02	Up head vs. body/tail
TLL1|7092	TLL1	7092	4.55	5.84	1.28	4.45	1.79E-05	1.54E-02	Up head vs. body/tail
RSPO3|84870	RSPO3	84870	4.87	6.42	1.55	4.42	2.00E-05	1.54E-02	Up head vs. body/tail
SORCS1|114815	SORCS1	114815	3.36	5.03	1.67	4.41	2.05E-05	1.54E-02	Up head vs. body/tail
CCL14|6358	CCL14	6358	5.79	7.53	1.74	4.40	2.12E-05	1.54E-02	Up head vs. body/tail
DNASE1L3|1776	DNASE1L3	1776	2.13	4.01	1.87	4.35	2.63E-05	1.71E-02	Up head vs. body/tail
PLP1|5354	PLP1	5354	4.06	6.02	1.96	4.32	3.02E-05	1.71E-02	Up head vs. body/tail
GFRA3|2676	GFRA3	2676	3.51	5.10	1.60	4.28	3.43E-05	1.71E-02	Up head vs. body/tail
PCK1|5105	PCK1	5105	3.95	6.00	2.05	4.24	4.00E-05	1.71E-02	Up head vs. body/tail
BEND4|389206	BEND4	389206	1.13	2.77	1.65	4.23	4.18E-05	1.71E-02	Up head vs. body/tail
CHRDL2|25884	CHRDL2	25884	3.70	5.84	2.14	4.23	4.20E-05	1.71E-02	Up head vs. body/tail
MYRIP|25924	MYRIP	25924	3.90	5.36	1.46	4.23	4.27E-05	1.71E-02	Up head vs. body/tail
P2RX1|5023	P2RX1	5023	4.88	6.41	1.53	4.22	4.41E-05	1.71E-02	Up head vs. body/tail
TRAT1|50852	TRAT1	50852	2.63	4.13	1.50	4.18	5.07E-05	1.84E-02	Up head vs. body/tail
PDK4|5166	PDK4	5166	9.44	10.64	1.21	4.16	5.58E-05	1.94E-02	Up head vs. body/tail
SELE|6401	SELE	6401	4.13	6.03	1.90	4.15	5.75E-05	1.94E-02	Up head vs. body/tail
CD8A|925	CD8A	925	6.25	7.54	1.28	4.12	6.48E-05	2.00E-02	Up head vs. body/tail
ANGPTL7|10218	ANGPTL7	10218	2.21	4.49	2.28	4.12	6.63E-05	2.00E-02	Up head vs. body/tail
ZNF831|128611	ZNF831	128611	3.49	5.10	1.61	4.11	6.90E-05	2.00E-02	Up head vs. body/tail
GPR171|29909	GPR171	29909	3.88	5.18	1.29	4.10	6.97E-05	2.00E-02	Up head vs. body/tail
CHL1|10752	CHL1	10752	5.32	6.78	1.46	4.08	7.55E-05	2.04E-02	Up head vs. body/tail
GZMA|3001	GZMA	3001	5.81	6.91	1.10	4.07	7.82E-05	2.04E-02	Up head vs. body/tail
CD247|919	CD247	919	5.60	6.70	1.10	4.05	8.39E-05	2.04E-02	Up head vs. body/tail
SOX10|6663	SOX10	6663	4.05	5.57	1.52	4.05	8.53E-05	2.04E-02	Up head vs. body/tail
VIT|5212	VIT	5212	1.76	3.46	1.70	4.04	8.92E-05	2.04E-02	Up head vs. body/tail
KLRK1|22914	KLRK1	22914	4.63	5.87	1.23	4.03	9.28E-05	2.04E-02	Up head vs. body/tail
PIK3R5|23533	PIK3R5	23533	6.94	7.96	1.02	4.01	9.80E-05	2.04E-02	Up head vs. body/tail
MADCAM1|8174	MADCAM1	8174	3.52	5.37	1.84	4.01	9.87E-05	2.04E-02	Up head vs. body/tail
CD48|962	CD48	962	6.78	8.04	1.26	4.00	1.03E-04	2.06E-02	Up head vs. body/tail

SH2D1A|4068	SH2D1A	4068	4.04	5.43	1.40	3.96	1.20E-04	2.27E-02	Up head vs. body/tail
CDH19|28513	CDH19	28513	3.27	4.84	1.57	3.96	1.21E-04	2.27E-02	Up head vs. body/tail
CD8B|926	CD8B	926	4.60	5.82	1.22	3.95	1.26E-04	2.27E-02	Up head vs. body/tail
BTLA|151888	BTLA	151888	2.68	4.24	1.56	3.94	1.27E-04	2.27E-02	Up head vs. body/tail
CD3D|915	CD3D	915	5.69	6.83	1.14	3.94	1.31E-04	2.27E-02	Up head vs. body/tail
CTSG|1511	CTSG	1511	2.82	4.55	1.73	3.90	1.49E-04	2.42E-02	Up head vs. body/tail
RBP5|83758	RBP5	83758	5.32	6.39	1.07	3.88	1.59E-04	2.49E-02	Up head vs. body/tail
CD2|914	CD2	914	6.76	7.88	1.12	3.87	1.67E-04	2.49E-02	Up head vs. body/tail
GZMB|3002	GZMB	3002	4.54	5.68	1.14	3.87	1.69E-04	2.49E-02	Up head vs. body/tail
CFP|5199	CFP	5199	4.83	6.01	1.18	3.85	1.80E-04	2.53E-02	Up head vs. body/tail
NGFR|4804	NGFR	4804	7.36	8.82	1.46	3.84	1.88E-04	2.53E-02	Up head vs. body/tail
VIPR2|7434	VIPR2	7434	3.12	4.81	1.69	3.84	1.90E-04	2.53E-02	Up head vs. body/tail
ZNF385D|79750	ZNF385D	79750	3.41	4.64	1.23	3.83	1.97E-04	2.54E-02	Up head vs. body/tail
CD36|948	CD36	948	7.27	8.62	1.34	3.81	2.06E-04	2.56E-02	Up head vs. body/tail
LYVE1|10894	LYVE1	10894	5.36	6.57	1.21	3.81	2.09E-04	2.56E-02	Up head vs. body/tail
FAM65B|9750	FAM65B	9750	5.63	6.94	1.32	3.79	2.21E-04	2.67E-02	Up head vs. body/tail
CNTN2|6900	CNTN2	6900	1.92	3.31	1.40	3.78	2.30E-04	2.75E-02	Up head vs. body/tail
CHRDL1|91851	CHRDL1	91851	6.19	7.99	1.80	3.78	2.36E-04	2.76E-02	Up head vs. body/tail
CXCR6|10663	CXCR6	10663	5.25	6.34	1.09	3.76	2.48E-04	2.76E-02	Up head vs. body/tail
PYHIN1|149628	PYHIN1	149628	3.21	4.60	1.39	3.76	2.54E-04	2.76E-02	Up head vs. body/tail
GPR18|2841	GPR18	2841	2.55	3.84	1.29	3.75	2.62E-04	2.76E-02	Up head vs. body/tail
CD3E|916	CD3E	916	7.07	8.18	1.11	3.71	3.03E-04	2.88E-02	Up head vs. body/tail
CD300LG|146894	CD300LG	146894	0.79	2.37	1.58	3.70	3.16E-04	2.93E-02	Up head vs. body/tail
NLRP6|171389	NLRP6	171389	1.86	2.91	1.04	3.67	3.49E-04	2.99E-02	Up head vs. body/tail
CARTPT|9607	CARTPT	9607	1.34	3.27	1.93	3.64	3.79E-04	2.99E-02	Up head vs. body/tail
SEPT1|1731	SEPT1	1731	6.09	7.16	1.07	3.64	3.81E-04	2.99E-02	Up head vs. body/tail
ASB2|51676	ASB2	51676	4.74	5.89	1.16	3.62	4.07E-04	2.99E-02	Up head vs. body/tail
KLRB1|3820	KLRB1	3820	5.00	6.16	1.15	3.62	4.13E-04	2.99E-02	Up head vs. body/tail
SCARA5|286133	SCARA5	286133	3.31	5.42	2.11	3.62	4.18E-04	2.99E-02	Up head vs. body/tail
ANGPTL1|9068	ANGPTL1	9068	5.73	7.00	1.27	3.61	4.35E-04	3.06E-02	Up head vs. body/tail
COL19A1|1310	COL19A1	1310	0.74	2.10	1.36	3.59	4.54E-04	3.14E-02	Up head vs. body/tail
CD96|10225	CD96	10225	5.78	6.87	1.09	3.58	4.69E-04	3.16E-02	Up head vs. body/tail
FITM1|161247	FITM1	161247	2.41	3.47	1.06	3.58	4.83E-04	3.23E-02	Up head vs. body/tail
ADH1B|125	ADH1B	125	7.07	9.00	1.92	3.57	4.92E-04	3.26E-02	Up head vs. body/tail
GPR97|222487	GPR97	222487	3.95	5.12	1.17	3.56	5.16E-04	3.32E-02	Up head vs. body/tail
P2RY14|9934	P2RY14	9934	3.97	5.04	1.06	3.54	5.38E-04	3.36E-02	Up head vs. body/tail
ZAP70|7535	ZAP70	7535	5.52	6.71	1.19	3.54	5.40E-04	3.36E-02	Up head vs. body/tail
THEMIS|387357	THEMIS	387357	3.30	4.59	1.29	3.53	5.58E-04	3.36E-02	Up head vs. body/tail
HPR|3250	HPR	3250	0.79	1.98	1.19	3.53	5.65E-04	3.36E-02	Up head vs. body/tail
FIGF|2277	FIGF	2277	1.77	2.95	1.19	3.53	5.65E-04	3.36E-02	Up head vs. body/tail

RHOH|399	RHOH	399	6.00	7.04	1.04	3.51	6.06E-04	3.46E-02	Up head vs. body/tail
GVIN1|387751	GVIN1	387751	5.33	6.46	1.12	3.51	6.10E-04	3.46E-02	Up head vs. body/tail
SFRP1|6422	SFRP1	6422	6.70	8.22	1.53	3.48	6.71E-04	3.59E-02	Up head vs. body/tail
IL24|11009	IL24	11009	1.40	2.47	1.07	3.48	6.76E-04	3.59E-02	Up head vs. body/tail
CADM3|57863	CADM3	57863	4.86	6.38	1.52	3.47	7.00E-04	3.70E-02	Up head vs. body/tail
PCDH8|5100	PCDH8	5100	0.96	2.17	1.21	3.46	7.30E-04	3.83E-02	Up head vs. body/tail
SLAMF1|6504	SLAMF1	6504	4.20	5.29	1.09	3.45	7.40E-04	3.83E-02	Up head vs. body/tail
CHI3L2|1117	CHI3L2	1117	4.42	5.62	1.20	3.44	7.77E-04	3.83E-02	Up head vs. body/tail
SLAMF6|114836	SLAMF6	114836	6.07	7.11	1.05	3.44	7.81E-04	3.83E-02	Up head vs. body/tail
CCL21|6366	CCL21	6366	7.31	9.06	1.75	3.43	8.01E-04	3.83E-02	Up head vs. body/tail
AOAH|313	AOAH	313	5.90	7.02	1.13	3.43	8.05E-04	3.83E-02	Up head vs. body/tail
NCF1B|654816	NCF1B	654816	4.06	5.12	1.06	3.43	8.06E-04	3.83E-02	Up head vs. body/tail
CCR7|1236	CCR7	1236	5.41	6.82	1.41	3.43	8.09E-04	3.83E-02	Up head vs. body/tail
FAIM3|9214	FAIM3	9214	6.46	7.69	1.24	3.43	8.10E-04	3.83E-02	Up head vs. body/tail
SCML4|256380	SCML4	256380	4.28	5.37	1.09	3.42	8.13E-04	3.83E-02	Up head vs. body/tail
PRKCB|5579	PRKCB	5579	6.17	7.42	1.25	3.42	8.28E-04	3.83E-02	Up head vs. body/tail
ABCA9|10350	ABCA9	10350	5.80	6.83	1.03	3.42	8.29E-04	3.83E-02	Up head vs. body/tail
CCR4|1233	CCR4	1233	3.40	4.74	1.35	3.41	8.47E-04	3.83E-02	Up head vs. body/tail
LOC283663|283663	LOC283663	283663	4.97	6.19	1.22	3.41	8.50E-04	3.83E-02	Up head vs. body/tail
VIP|7432	VIP	7432	3.71	5.67	1.96	3.40	8.83E-04	3.87E-02	Up head vs. body/tail
ADH1A|124	ADH1A	124	2.39	3.77	1.38	3.40	8.94E-04	3.89E-02	Up head vs. body/tail
HLA-DQA1|3117	HLA-DQA1	3117	9.68	10.76	1.09	3.39	9.11E-04	3.92E-02	Up head vs. body/tail
LTB|4050	LTB	4050	7.29	8.39	1.10	3.38	9.32E-04	3.97E-02	Up head vs. body/tail
XPNPEP2|7512	XPNPEP2	7512	2.45	4.04	1.59	3.38	9.39E-04	3.97E-02	Up head vs. body/tail
GHRL|51738	GHRL	51738	3.51	5.18	1.67	3.38	9.49E-04	3.97E-02	Up head vs. body/tail
STAB2|55576	STAB2	55576	1.54	2.87	1.33	3.37	9.61E-04	4.00E-02	Up head vs. body/tail
LY9|4063	LY9	4063	4.27	5.48	1.21	3.36	9.96E-04	4.07E-02	Up head vs. body/tail
RBP2|5948	RBP2	5948	1.11	2.70	1.59	3.36	1.01E-03	4.10E-02	Up head vs. body/tail
SIT1|27240	SIT1	27240	4.16	5.38	1.21	3.35	1.03E-03	4.15E-02	Up head vs. body/tail
CNR1|1268	CNR1	1268	4.77	5.94	1.17	3.35	1.05E-03	4.19E-02	Up head vs. body/tail
PMP2|5375	PMP2	5375	1.84	3.23	1.39	3.35	1.06E-03	4.20E-02	Up head vs. body/tail
ICOS|29851	ICOS	29851	3.64	4.68	1.04	3.34	1.07E-03	4.20E-02	Up head vs. body/tail
SLC1A2|6506	SLC1A2	6506	3.75	5.15	1.40	3.34	1.08E-03	4.20E-02	Up head vs. body/tail
FAM107A|11170	FAM107A	11170	6.74	7.74	1.00	3.33	1.12E-03	4.25E-02	Up head vs. body/tail
PGM5|5239	PGM5	5239	6.29	7.36	1.07	3.32	1.15E-03	4.25E-02	Up head vs. body/tail
PDCD1|5133	PDCD1	5133	4.24	5.25	1.01	3.32	1.16E-03	4.25E-02	Up head vs. body/tail
CLEC10A|10462	CLEC10A	10462	5.25	6.49	1.25	3.31	1.18E-03	4.26E-02	Up head vs. body/tail
CD40LG|959	CD40LG	959	3.11	4.24	1.13	3.31	1.19E-03	4.26E-02	Up head vs. body/tail
TLR10|81793	TLR10	81793	3.49	4.79	1.31	3.30	1.22E-03	4.27E-02	Up head vs. body/tail
ITK|3702	ITK	3702	5.47	6.67	1.20	3.30	1.22E-03	4.28E-02	Up head vs. body/tail

MAP4K1|11184	MAP4K1	11184	5.95	7.02	1.06	3.29	1.27E-03	4.39E-02	Up head vs. body/tail
PI16|221476	PI16	221476	4.10	6.02	1.91	3.29	1.27E-03	4.39E-02	Up head vs. body/tail
AOX1|316	AOX1	316	6.58	7.93	1.34	3.28	1.30E-03	4.45E-02	Up head vs. body/tail
LCT|3938	LCT	3938	0.18	1.61	1.42	3.26	1.39E-03	4.62E-02	Up head vs. body/tail
SLIT3|6586	SLIT3	6586	8.24	9.27	1.03	3.25	1.47E-03	4.77E-02	Up head vs. body/tail
P2RY10|27334	P2RY10	27334	3.55	4.70	1.15	3.24	1.52E-03	4.84E-02	Up head vs. body/tail
NCF1C|654817	NCF1C	654817	4.31	5.39	1.08	3.23	1.56E-03	4.88E-02	Up head vs. body/tail
CD79B|974	CD79B	974	5.46	6.83	1.37	3.22	1.59E-03	4.92E-02	Up head vs. body/tail
PLIN1|5346	PLIN1	5346	3.08	4.95	1.88	3.21	1.64E-03	4.98E-02	Up head vs. body/tail
FABP4|2167	FABP4	2167	5.08	6.84	1.76	3.21	1.66E-03	4.98E-02	Up head vs. body/tail
IRF4|3662	IRF4	3662	5.11	6.22	1.11	3.19	1.77E-03	5.12E-02	Up head vs. body/tail
CXCR2|3579	CXCR2	3579	3.27	4.51	1.24	3.19	1.78E-03	5.13E-02	Up head vs. body/tail
DES|1674	DES	1674	5.93	8.04	2.10	3.18	1.80E-03	5.15E-02	Up head vs. body/tail
ZBTB16|7704	ZBTB16	7704	5.16	6.52	1.35	3.18	1.81E-03	5.15E-02	Up head vs. body/tail
KIAA1324L|222223	KIAA1324L	222223	6.06	7.07	1.01	3.16	1.95E-03	5.26E-02	Up head vs. body/tail
CCL19|6363	CCL19	6363	6.50	8.33	1.83	3.15	1.97E-03	5.28E-02	Up head vs. body/tail
GNMT|27232	GNMT	27232	2.61	4.31	1.69	3.15	1.98E-03	5.28E-02	Up head vs. body/tail
C13orf33|84935	C13orf33	84935	7.17	8.32	1.15	3.15	2.00E-03	5.28E-02	Up head vs. body/tail
CR1|1378	CR1	1378	5.41	6.71	1.29	3.15	2.02E-03	5.28E-02	Up head vs. body/tail
CXCL13|10563	CXCL13	10563	4.38	6.44	2.07	3.14	2.06E-03	5.29E-02	Up head vs. body/tail
GP1BA|2811	GP1BA	2811	2.77	3.97	1.19	3.13	2.14E-03	5.39E-02	Up head vs. body/tail
PI15|51050	PI15	51050	6.47	7.57	1.10	3.13	2.15E-03	5.40E-02	Up head vs. body/tail
RSPO1|284654	RSPO1	284654	2.72	3.82	1.10	3.13	2.17E-03	5.42E-02	Up head vs. body/tail
PTPRC|5788	PTPRC	5788	8.79	9.81	1.02	3.11	2.31E-03	5.54E-02	Up head vs. body/tail
GZMK|3003	GZMK	3003	5.32	6.55	1.24	3.07	2.61E-03	5.77E-02	Up head vs. body/tail
PRIMA1|145270	PRIMA1	145270	4.16	5.29	1.13	3.07	2.61E-03	5.77E-02	Up head vs. body/tail
ADAMTS8|11095	ADAMTS8	11095	5.18	6.42	1.24	3.06	2.69E-03	5.87E-02	Up head vs. body/tail
PTF1A|256297	PTF1A	256297	0.76	2.70	1.94	3.03	2.91E-03	6.12E-02	Up head vs. body/tail
CD3G|917	CD3G	917	3.60	4.61	1.01	3.03	2.92E-03	6.12E-02	Up head vs. body/tail
RASGRP2|10235	RASGRP2	10235	6.15	7.17	1.02	3.00	3.24E-03	6.50E-02	Up head vs. body/tail
MYOCD|93649	MYOCD	93649	3.26	4.42	1.16	2.98	3.40E-03	6.70E-02	Up head vs. body/tail
CXCR5|643	CXCR5	643	3.59	5.07	1.48	2.98	3.45E-03	6.70E-02	Up head vs. body/tail
ABCA8|10351	ABCA8	10351	6.45	7.58	1.13	2.95	3.78E-03	6.86E-02	Up head vs. body/tail
TCL1A|8115	TCL1A	8115	1.90	3.93	2.04	2.94	3.80E-03	6.86E-02	Up head vs. body/tail
CAPN6|827	CAPN6	827	7.17	8.25	1.08	2.94	3.87E-03	6.91E-02	Up head vs. body/tail
REG1P|5969	REG1P	5969	1.77	4.47	2.70	2.93	3.95E-03	6.94E-02	Up head vs. body/tail
LMO3|55885	LMO3	55885	5.38	6.39	1.01	2.92	4.06E-03	6.97E-02	Up head vs. body/tail
MT1A|4489	MT1A	4489	4.01	5.15	1.15	2.92	4.07E-03	6.97E-02	Up head vs. body/tail
KLHL14|57565	KLHL14	57565	1.91	3.05	1.14	2.92	4.14E-03	7.01E-02	Up head vs. body/tail
POU2AF1|5450	POU2AF1	5450	6.30	7.49	1.20	2.91	4.24E-03	7.07E-02	Up head vs. body/tail

CUZD1|50624	CUZD1	50624	5.80	8.11	2.32	2.91	4.24E-03	7.07E-02	Up head vs. body/tail
PENK|5179	PENK	5179	2.89	4.08	1.20	2.91	4.27E-03	7.09E-02	Up head vs. body/tail
ADAMDEC1|27299	ADAMDEC1	27299	4.67	5.77	1.10	2.90	4.31E-03	7.09E-02	Up head vs. body/tail
CLEC4E|26253	CLEC4E	26253	4.48	5.51	1.02	2.90	4.35E-03	7.11E-02	Up head vs. body/tail
SLC26A3|1811	SLC26A3	1811	0.44	2.19	1.74	2.89	4.50E-03	7.26E-02	Up head vs. body/tail
APOA4|337	APOA4	337	0.76	2.86	2.09	2.87	4.69E-03	7.31E-02	Up head vs. body/tail
BLK|640	BLK	640	2.92	4.65	1.73	2.87	4.72E-03	7.33E-02	Up head vs. body/tail
CTRL|1506	CTRL	1506	4.64	7.14	2.50	2.87	4.81E-03	7.40E-02	Up head vs. body/tail
MT1H|4496	MT1H	4496	3.54	4.83	1.29	2.85	4.98E-03	7.52E-02	Up head vs. body/tail
STMN4|81551	STMN4	81551	0.98	2.16	1.18	2.85	5.07E-03	7.63E-02	Up head vs. body/tail
SERPINI2|5276	SERPINI2	5276	2.16	4.84	2.68	2.84	5.13E-03	7.69E-02	Up head vs. body/tail
MYBPC2|4606	MYBPC2	4606	1.63	2.63	1.00	2.84	5.15E-03	7.70E-02	Up head vs. body/tail
ADH4|127	ADH4	127	1.75	3.02	1.27	2.82	5.46E-03	7.90E-02	Up head vs. body/tail
GUCA1C|9626	GUCA1C	9626	0.25	1.72	1.47	2.82	5.54E-03	7.96E-02	Up head vs. body/tail
IGF1|3479	IGF1	3479	6.72	8.03	1.31	2.82	5.57E-03	7.97E-02	Up head vs. body/tail
TNFRSF13B|23495	TNFRSF13B	23495	1.77	3.10	1.32	2.81	5.63E-03	7.99E-02	Up head vs. body/tail
C16orf54|283897	C16orf54	283897	5.75	6.79	1.03	2.81	5.72E-03	8.08E-02	Up head vs. body/tail
CNTFR|1271	CNTFR	1271	2.19	3.62	1.42	2.81	5.74E-03	8.09E-02	Up head vs. body/tail
FCRL1|115350	FCRL1	115350	1.58	3.21	1.62	2.80	5.84E-03	8.13E-02	Up head vs. body/tail
RELN|5649	RELN	5649	4.87	6.21	1.33	2.78	6.14E-03	8.27E-02	Up head vs. body/tail
C16orf89|146556	C16orf89	146556	5.17	6.32	1.16	2.78	6.23E-03	8.32E-02	Up head vs. body/tail
OGN|4969	OGN	4969	6.74	8.00	1.26	2.77	6.32E-03	8.34E-02	Up head vs. body/tail
SLC7A9|11136	SLC7A9	11136	2.26	3.28	1.02	2.77	6.33E-03	8.34E-02	Up head vs. body/tail
CCL23|6368	CCL23	6368	2.62	3.73	1.12	2.76	6.59E-03	8.47E-02	Up head vs. body/tail
LOC284749|284749	LOC284749	284749	2.31	3.42	1.10	2.76	6.60E-03	8.47E-02	Up head vs. body/tail
PLA2G2A|5320	PLA2G2A	5320	5.53	7.45	1.92	2.75	6.76E-03	8.57E-02	Up head vs. body/tail
MT1G|4495	MT1G	4495	7.23	8.44	1.21	2.73	7.22E-03	8.79E-02	Up head vs. body/tail
MS4A1|931	MS4A1	931	3.83	5.83	2.00	2.72	7.27E-03	8.83E-02	Up head vs. body/tail
ATP1A2|477	ATP1A2	477	4.16	5.32	1.16	2.72	7.28E-03	8.83E-02	Up head vs. body/tail
CYP4F2|8529	CYP4F2	8529	1.23	2.52	1.29	2.72	7.30E-03	8.83E-02	Up head vs. body/tail
FAM129C|199786	FAM129C	199786	1.92	3.46	1.54	2.70	7.77E-03	9.00E-02	Up head vs. body/tail
LCN10|414332	LCN10	414332	3.39	4.42	1.03	2.69	7.92E-03	9.03E-02	Up head vs. body/tail
REG1B|5968	REG1B	5968	5.26	8.62	3.36	2.69	8.04E-03	9.09E-02	Up head vs. body/tail
STAP1|26228	STAP1	26228	2.60	3.75	1.15	2.68	8.15E-03	9.13E-02	Up head vs. body/tail
CELP|1057	CELP	1057	1.80	4.88	3.08	2.68	8.37E-03	9.18E-02	Up head vs. body/tail
CNR2|1269	CNR2	1269	1.30	2.59	1.28	2.67	8.53E-03	9.29E-02	Up head vs. body/tail
FCRLA|84824	FCRLA	84824	2.95	4.46	1.51	2.66	8.70E-03	9.35E-02	Up head vs. body/tail
ADIPOQ|9370	ADIPOQ	9370	1.64	3.92	2.27	2.65	8.88E-03	9.39E-02	Up head vs. body/tail
AQP8|343	AQP8	343	2.40	4.82	2.43	2.64	9.27E-03	9.55E-02	Up head vs. body/tail
CLEC17A|388512	CLEC17A	388512	1.63	2.86	1.22	2.64	9.37E-03	9.58E-02	Up head vs. body/tail

IGFN1|91156	IGFN1	91156	3.09	4.21	1.13	2.62	9.81E-03	9.74E-02	Up head vs. body/tail
AMY2B|280	AMY2B	280	7.88	9.68	1.81	2.62	9.83E-03	9.74E-02	Up head vs. body/tail
GPHA2|170589	GPHA2	170589	1.03	2.67	1.64	2.61	9.95E-03	9.81E-02	Up head vs. body/tail
LGALS2|3957	LGALS2	3957	6.30	7.53	1.23	2.61	1.00E-02	9.82E-02	Up head vs. body/tail
ERP27|121506	ERP27	121506	6.41	7.86	1.45	2.61	1.01E-02	9.84E-02	Up head vs. body/tail
FCER2|2208	FCER2	2208	1.94	3.57	1.62	2.54	1.22E-02	1.07E-01	Up head vs. body/tail
CD19|930	CD19	930	2.66	4.25	1.60	2.54	1.23E-02	1.07E-01	Up head vs. body/tail
APOA1|335	APOA1	335	2.14	3.60	1.46	2.53	1.24E-02	1.08E-01	Up head vs. body/tail
ACTG2|72	ACTG2	72	8.15	9.32	1.17	2.51	1.32E-02	1.11E-01	Up head vs. body/tail
CSF3|1440	CSF3	1440	1.14	2.38	1.24	2.50	1.36E-02	1.12E-01	Up head vs. body/tail
FPR2|2358	FPR2	2358	3.77	4.85	1.08	2.50	1.37E-02	1.12E-01	Up head vs. body/tail
CELA3B|23436	CELA3B	23436	4.86	8.16	3.29	2.50	1.37E-02	1.12E-01	Up head vs. body/tail
LOC220594|220594	LOC220594	220594	4.66	5.71	1.05	2.50	1.37E-02	1.12E-01	Up head vs. body/tail
GP2|2813	GP2	2813	6.89	9.85	2.96	2.50	1.37E-02	1.12E-01	Up head vs. body/tail
SI|6476	SI	6476	0.93	2.77	1.83	2.49	1.39E-02	1.13E-01	Up head vs. body/tail
FCRL3|115352	FCRL3	115352	3.05	4.26	1.21	2.49	1.41E-02	1.13E-01	Up head vs. body/tail
CELA2B|51032	CELA2B	51032	3.17	6.17	3.00	2.48	1.43E-02	1.14E-01	Up head vs. body/tail
KCNJ5|3762	KCNJ5	3762	4.57	5.58	1.01	2.47	1.48E-02	1.16E-01	Up head vs. body/tail
ARHGDIG|398	ARHGDIG	398	2.82	4.04	1.22	2.45	1.56E-02	1.20E-01	Up head vs. body/tail
SLC39A5|283375	SLC39A5	283375	5.68	7.17	1.49	2.44	1.60E-02	1.21E-01	Up head vs. body/tail
REG3G|130120	REG3G	130120	4.03	6.80	2.77	2.43	1.62E-02	1.22E-01	Up head vs. body/tail
SIGLEC14|100049587	SIGLEC14	100049587	4.60	5.64	1.04	2.43	1.62E-02	1.22E-01	Up head vs. body/tail
PNLIPRP1|5407	PNLIPRP1	5407	3.90	6.82	2.92	2.43	1.64E-02	1.22E-01	Up head vs. body/tail
FBP2|8789	FBP2	8789	1.14	2.20	1.06	2.42	1.70E-02	1.24E-01	Up head vs. body/tail
PAX5|5079	PAX5	5079	1.86	3.20	1.33	2.42	1.70E-02	1.24E-01	Up head vs. body/tail
ITIH4|3700	ITIH4	3700	6.74	7.81	1.07	2.41	1.71E-02	1.24E-01	Up head vs. body/tail
CTRC|11330	CTRC	11330	5.60	8.69	3.10	2.41	1.72E-02	1.25E-01	Up head vs. body/tail
C7|730	C7	730	10.88	11.95	1.07	2.41	1.74E-02	1.25E-01	Up head vs. body/tail
FABP1|2168	FABP1	2168	1.84	3.53	1.69	2.39	1.80E-02	1.27E-01	Up head vs. body/tail
CEL|1056	CEL	1056	7.52	10.29	2.77	2.39	1.83E-02	1.28E-01	Up head vs. body/tail
KLB|152831	KLB	152831	2.56	3.58	1.02	2.39	1.84E-02	1.28E-01	Up head vs. body/tail
PTX3|5806	PTX3	5806	4.95	5.96	1.01	2.37	1.90E-02	1.30E-01	Up head vs. body/tail
ALDOB|229	ALDOB	229	7.88	8.97	1.09	2.37	1.92E-02	1.31E-01	Up head vs. body/tail
RBPJL|11317	RBPJL	11317	2.01	4.09	2.08	2.37	1.94E-02	1.31E-01	Up head vs. body/tail
LBP|3929	LBP	3929	2.06	3.45	1.39	2.36	1.96E-02	1.32E-01	Up head vs. body/tail
CPA2|1358	CPA2	1358	5.67	8.71	3.04	2.36	1.98E-02	1.32E-01	Up head vs. body/tail
REG3A|5068	REG3A	5068	7.25	10.05	2.81	2.35	2.04E-02	1.35E-01	Up head vs. body/tail
OTC|5009	OTC	5009	1.65	2.89	1.25	2.34	2.08E-02	1.36E-01	Up head vs. body/tail
AMY2A|279	AMY2A	279	5.19	8.22	3.03	2.33	2.13E-02	1.38E-01	Up head vs. body/tail
PLA2G2D|26279	PLA2G2D	26279	3.70	4.87	1.17	2.33	2.14E-02	1.38E-01	Up head vs. body/tail

PRPH|5630	PRPH	5630	4.23	5.37	1.14	2.32	2.16E-02	1.39E-01	Up head vs. body/tail
TRIM50|135892	TRIM50	135892	1.91	3.04	1.13	2.31	2.24E-02	1.41E-01	Up head vs. body/tail
CYP3A4|1576	CYP3A4	1576	2.26	3.47	1.21	2.30	2.31E-02	1.44E-01	Up head vs. body/tail
MEP1A|4224	MEP1A	4224	1.89	3.19	1.30	2.30	2.32E-02	1.44E-01	Up head vs. body/tail
PLIN4|729359	PLIN4	729359	6.01	7.27	1.26	2.30	2.32E-02	1.44E-01	Up head vs. body/tail
GSTA2|2939	GSTA2	2939	3.70	5.11	1.41	2.29	2.33E-02	1.44E-01	Up head vs. body/tail
PPY|5539	PPY	5539	6.80	9.08	2.28	2.29	2.35E-02	1.45E-01	Up head vs. body/tail
CR2|1380	CR2	1380	3.84	5.86	2.02	2.29	2.35E-02	1.45E-01	Up head vs. body/tail
BHLHA15|168620	BHLHA15	168620	2.63	3.77	1.13	2.29	2.38E-02	1.45E-01	Up head vs. body/tail
PLA2G1B|5319	PLA2G1B	5319	5.16	7.95	2.79	2.28	2.44E-02	1.46E-01	Up head vs. body/tail
LOC723809|723809	LOC723809	723809	3.26	4.34	1.08	2.26	2.52E-02	1.48E-01	Up head vs. body/tail
CCL15|6359	CCL15	6359	4.85	5.98	1.12	2.25	2.59E-02	1.50E-01	Up head vs. body/tail
PM20D1|148811	PM20D1	148811	2.46	3.66	1.19	2.24	2.66E-02	1.52E-01	Up head vs. body/tail
GABRP|2568	GABRP	2568	9.55	10.82	1.27	2.24	2.70E-02	1.53E-01	Up head vs. body/tail
AMY1A|276	AMY1A	276	2.39	4.00	1.60	2.23	2.77E-02	1.54E-01	Up head vs. body/tail
SPINK1|6690	SPINK1	6690	11.44	12.64	1.20	2.22	2.79E-02	1.55E-01	Up head vs. body/tail
NPHS1|4868	NPHS1	4868	3.16	4.35	1.19	2.21	2.90E-02	1.58E-01	Up head vs. body/tail
APOB|338	APOB	338	3.38	4.52	1.14	2.18	3.11E-02	1.63E-01	Up head vs. body/tail
LEP|3952	LEP	3952	1.86	3.14	1.28	2.18	3.11E-02	1.63E-01	Up head vs. body/tail
DUSP27|92235	DUSP27	92235	1.71	3.14	1.42	2.17	3.17E-02	1.64E-01	Up head vs. body/tail
SYCE1|93426	SYCE1	93426	1.97	3.23	1.25	2.16	3.22E-02	1.65E-01	Up head vs. body/tail
SYCN|342898	SYCN	342898	3.49	5.99	2.50	2.16	3.26E-02	1.66E-01	Up head vs. body/tail
CIDEA|1149	CIDEA	1149	0.56	1.74	1.18	2.15	3.29E-02	1.67E-01	Up head vs. body/tail
CELA3A|10136	CELA3A	10136	6.85	9.84	2.99	2.15	3.30E-02	1.67E-01	Up head vs. body/tail
CPA1|1357	CPA1	1357	7.36	10.29	2.94	2.14	3.40E-02	1.69E-01	Up head vs. body/tail
MYBPC1|4604	MYBPC1	4604	2.39	4.11	1.71	2.13	3.49E-02	1.71E-01	Up head vs. body/tail
BTNL3|10917	BTNL3	10917	2.28	3.72	1.44	2.13	3.50E-02	1.71E-01	Up head vs. body/tail
NEB|4703	NEB	4703	5.66	6.67	1.01	2.12	3.58E-02	1.74E-01	Up head vs. body/tail
CPB1|1360	CPB1	1360	8.07	10.82	2.76	2.12	3.59E-02	1.74E-01	Up head vs. body/tail
FCN1|2219	FCN1	2219	5.48	6.52	1.04	2.12	3.61E-02	1.74E-01	Up head vs. body/tail
CELA2A|63036	CELA2A	63036	4.68	7.63	2.95	2.11	3.67E-02	1.75E-01	Up head vs. body/tail
FCGR3B|2215	FCGR3B	2215	4.25	5.30	1.04	2.10	3.75E-02	1.78E-01	Up head vs. body/tail
FGL1|2267	FGL1	2267	4.07	5.56	1.49	2.10	3.79E-02	1.78E-01	Up head vs. body/tail
HMGCS2|3158	HMGCS2	3158	5.83	7.29	1.46	2.09	3.82E-02	1.78E-01	Up head vs. body/tail
TUSC5|286753	TUSC5	286753	1.41	2.70	1.28	2.09	3.87E-02	1.79E-01	Up head vs. body/tail
CTRB2|440387	CTRB2	440387	7.62	10.55	2.93	2.08	3.91E-02	1.80E-01	Up head vs. body/tail
GATA5|140628	GATA5	140628	1.37	2.52	1.15	2.04	4.28E-02	1.86E-01	Up head vs. body/tail
AQP12A|375318	AQP12A	375318	0.63	1.69	1.06	2.02	4.49E-02	1.90E-01	Up head vs. body/tail
PDIA2|64714	PDIA2	64714	5.70	7.35	1.65	2.02	4.55E-02	1.91E-01	Up head vs. body/tail
PNLIP|5406	PNLIP	5406	6.45	9.41	2.96	2.02	4.57E-02	1.91E-01	Up head vs. body/tail

KIAA0125|9834	KIAA0125	9834	3.31	4.32	1.01	2.00	4.73E-02	1.93E-01	Up head vs. body/tail
KLK1|3816	KLK1	3816	7.12	8.56	1.45	2.00	4.76E-02	1.93E-01	Up head vs. body/tail
REG1A|5967	REG1A	5967	10.58	12.52	1.94	1.98	4.95E-02	1.96E-01	Up head vs. body/tail
CALY|50632	CALY	50632	5.71	4.65	-1.06	-1.98	4.93E-02	1.96E-01	Down head vs. body/tail
DNAJB13|374407	DNAJB13	374407	5.37	4.37	-1.00	-1.99	4.87E-02	1.95E-01	Down head vs. body/tail
CHGB|1114	CHGB	1114	11.38	10.14	-1.24	-1.99	4.86E-02	1.95E-01	Down head vs. body/tail
LGALS7|3963	LGALS7	3963	4.70	3.46	-1.24	-2.02	4.49E-02	1.90E-01	Down head vs. body/tail
PCSK2|5126	PCSK2	5126	8.89	7.54	-1.35	-2.06	4.17E-02	1.84E-01	Down head vs. body/tail
CPLX2|10814	CPLX2	10814	7.96	6.71	-1.25	-2.06	4.10E-02	1.84E-01	Down head vs. body/tail
KLK5|25818	KLK5	25818	3.70	2.39	-1.31	-2.07	4.07E-02	1.83E-01	Down head vs. body/tail
SYT8|90019	SYT8	90019	10.77	9.75	-1.01	-2.07	4.03E-02	1.82E-01	Down head vs. body/tail
LY6K|54742	LY6K	54742	5.34	4.05	-1.29	-2.09	3.85E-02	1.79E-01	Down head vs. body/tail
MUC21|394263	MUC21	394263	3.74	2.54	-1.20	-2.11	3.64E-02	1.75E-01	Down head vs. body/tail
FAM83A|84985	FAM83A	84985	9.64	8.52	-1.12	-2.11	3.63E-02	1.75E-01	Down head vs. body/tail
GRM4|2914	GRM4	2914	5.02	3.98	-1.03	-2.12	3.56E-02	1.73E-01	Down head vs. body/tail
TMEM40|55287	TMEM40	55287	4.33	3.23	-1.10	-2.14	3.41E-02	1.70E-01	Down head vs. body/tail
CRH|1392	CRH	1392	3.73	2.55	-1.18	-2.16	3.25E-02	1.66E-01	Down head vs. body/tail
SPRR2A|6700	SPRR2A	6700	3.47	2.37	-1.10	-2.21	2.86E-02	1.57E-01	Down head vs. body/tail
KRT16|3868	KRT16	3868	10.83	9.70	-1.13	-2.23	2.73E-02	1.54E-01	Down head vs. body/tail
KCNK16|83795	KCNK16	83795	6.85	5.37	-1.48	-2.24	2.70E-02	1.53E-01	Down head vs. body/tail
CHGA|1113	CHGA	1113	11.06	9.57	-1.50	-2.25	2.57E-02	1.49E-01	Down head vs. body/tail
UPK2|7379	UPK2	7379	4.01	2.80	-1.21	-2.26	2.56E-02	1.49E-01	Down head vs. body/tail
PIK3C2G|5288	PIK3C2G	5288	7.22	5.96	-1.26	-2.28	2.40E-02	1.45E-01	Down head vs. body/tail
CDKN2A|1029	CDKN2A	1029	7.87	6.66	-1.21	-2.30	2.31E-02	1.44E-01	Down head vs. body/tail
UGT1A9|54600	UGT1A9	54600	6.97	5.88	-1.09	-2.31	2.23E-02	1.41E-01	Down head vs. body/tail
LOC554202|554202	LOC554202	554202	6.74	5.55	-1.18	-2.36	1.97E-02	1.32E-01	Down head vs. body/tail
HAPLN1|1404	HAPLN1	1404	5.37	4.28	-1.09	-2.37	1.91E-02	1.30E-01	Down head vs. body/tail
TH|7054	TH	7054	3.20	2.17	-1.04	-2.38	1.88E-02	1.29E-01	Down head vs. body/tail
MSLN|10232	MSLN	10232	13.15	12.04	-1.11	-2.38	1.86E-02	1.29E-01	Down head vs. body/tail
CYP2W1|54905	CYP2W1	54905	6.37	5.23	-1.14	-2.41	1.71E-02	1.24E-01	Down head vs. body/tail
PNCK|139728	PNCK	139728	4.54	3.18	-1.36	-2.42	1.68E-02	1.24E-01	Down head vs. body/tail
FIBCD1|84929	FIBCD1	84929	7.05	5.68	-1.37	-2.46	1.49E-02	1.17E-01	Down head vs. body/tail
CGB7|94027	CGB7	94027	5.46	4.26	-1.20	-2.47	1.46E-02	1.16E-01	Down head vs. body/tail
CACNG6|59285	CACNG6	59285	4.60	3.14	-1.46	-2.48	1.43E-02	1.14E-01	Down head vs. body/tail
TNNT1|7138	TNNT1	7138	7.17	5.62	-1.54	-2.49	1.39E-02	1.13E-01	Down head vs. body/tail
CGB8|94115	CGB8	94115	4.95	3.57	-1.37	-2.53	1.25E-02	1.08E-01	Down head vs. body/tail
PKP1|5317	PKP1	5317	8.62	7.57	-1.05	-2.53	1.25E-02	1.08E-01	Down head vs. body/tail
SPRR1B|6699	SPRR1B	6699	7.13	5.33	-1.80	-2.54	1.21E-02	1.07E-01	Down head vs. body/tail
NTSR1|4923	NTSR1	4923	7.43	5.92	-1.51	-2.56	1.14E-02	1.04E-01	Down head vs. body/tail
WNT7A|7476	WNT7A	7476	8.27	6.92	-1.35	-2.58	1.09E-02	1.02E-01	Down head vs. body/tail

CLIC3|9022	CLIC3	9022	9.57	8.47	-1.10	-2.69	7.92E-03	9.03E-02	Down head vs. body/tail
NTF4|4909	NTF4	4909	4.85	3.71	-1.14	-2.71	7.51E-03	8.89E-02	Down head vs. body/tail
VGF|7425	VGF	7425	7.71	6.42	-1.29	-2.76	6.59E-03	8.47E-02	Down head vs. body/tail
SPRR3|6707	SPRR3	6707	7.81	5.83	-1.99	-2.79	6.01E-03	8.20E-02	Down head vs. body/tail
TTR|7276	TTR	7276	12.98	10.64	-2.34	-2.82	5.45E-03	7.90E-02	Down head vs. body/tail
EEF1A2|1917	EEF1A2	1917	9.87	8.84	-1.04	-2.87	4.69E-03	7.31E-02	Down head vs. body/tail
COL17A1|1308	COL17A1	1308	12.92	11.57	-1.35	-2.92	4.09E-03	6.98E-02	Down head vs. body/tail
FAM81B|153643	FAM81B	153643	5.38	4.21	-1.17	-2.97	3.54E-03	6.73E-02	Down head vs. body/tail
CRYBA2|1412	CRYBA2	1412	6.83	4.92	-1.91	-3.06	2.69E-03	5.87E-02	Down head vs. body/tail
CREG2|200407	CREG2	200407	5.77	4.69	-1.08	-3.14	2.06E-03	5.29E-02	Down head vs. body/tail
RTBDN|83546	RTBDN	83546	4.08	2.78	-1.31	-3.17	1.90E-03	5.20E-02	Down head vs. body/tail
TNS4|84951	TNS4	84951	11.50	10.23	-1.26	-3.23	1.55E-03	4.88E-02	Down head vs. body/tail
CFC1B|653275	CFC1B	653275	8.49	6.15	-2.35	-3.34	1.09E-03	4.20E-02	Down head vs. body/tail
BARX1|56033	BARX1	56033	4.93	3.22	-1.72	-3.45	7.53E-04	3.83E-02	Down head vs. body/tail
GCG|2641	GCG	2641	13.85	10.32	-3.52	-3.50	6.18E-04	3.47E-02	Down head vs. body/tail
C8orf73|642475	C8orf73	642475	10.34	9.26	-1.08	-3.93	1.32E-04	2.27E-02	Down head vs. body/tail
CYP26A1|1592	CYP26A1	1592	5.37	3.50	-1.87	-4.02	9.56E-05	2.04E-02	Down head vs. body/tail
GALNT5|11227	GALNT5	11227	10.23	9.03	-1.21	-4.39	2.21E-05	1.54E-02	Down head vs. body/tail
CASP14|23581	CASP14	23581	2.94	0.75	-2.19	-4.51	1.38E-05	1.54E-02	Down head vs. body/tail

Table S3. Ontology analysis of the 334 genes differentially expressed between the head and B/T location of PDAC. Ontologies were obtained using the DAVID Bioinformatics Resources 6.8 pathway analysis software.

				up Head vs. Body/Tail				down Head vs. Body/Tail		
**Base**	**TermsID**	**Terms**	**N**	**Genes**	**p-value**	**q-value**	**N**	**Genes**	**p-value**	**q-value**
GO_BP	GO:0006955	immune response	30	GZMH,CCL14,CD8A,GZMA,MADCAM1,CD8B,CTSG,CFP,NGFR,CD96,ZAP70,IL24,CCL21,CCR7,CCR4,HLA-DQA1,LTB,ICOS,TLR10,CD79B,CCL19,CXCL13,CXCR5,ADAMDEC1,CCL23,CNR2,CSF3,CR2,CCL15,FCGR3B	2.10883E-15	1.07129E-12				
GO_BP	GO:0007186	G-protein coupled receptor signaling pathway	27	ACKR1,CCL14,GPR171,PIK3R5,VIPR2,CXCR6,GPR18,CD3E,NLRP6,CARTPT,GPR97,P2RY14,CCL21,CCR7,CCR4,VIP,GHRL,CXCR2,CCL19,CXCL13,CXCR5,CCL23,APOA1,FPR2,BHLHA15,CCL15,FCN1	2.21932E-12	5.63707E-10				

GO_BP	GO:0006954	inflammatory response	23	ACKR1,SELP,PLP1,SELE,NGFR,CXCR6,NLRP6,IL24,CCL21,AOAH,CCR7,CCR4,CD40LG,TLR10,AOX1,CXCR2,CCL19,CXCL13,CCL23,CNR2,FPR2,PTX3,PLA2G2D	1.58706E-09	2.68742E-07
GO_BP	GO:0002250	adaptive immune response	12	TRAT1,KLRK1,CD8B,BTLA,ZAP70,THEMIS,PRKCB,SIT1,CLEC10A,ITK,CD79B,TNFRSF13B	3.08526E-08	3.91827E-06
GO_BP	GO:0019433	triglyceride catabolic process	6	PLIN1,FABP4,APOA4,APOA1,FABP1,APOB	2.31913E-07	2.35624E-05
GO_BP	GO:0007166	cell surface receptor signaling pathway	16	GFRA1,CD8A,CD3D,CD2,VIPR2,CD36,CD3E,KLRB1,GPR97,CXCR2,CXCL13,GP1BA,PTPRC,CD3G,TNFRSF13B,CD19	8.65374E-07	6.63635E-05
GO_BP	GO:0006968	cellular defense response	7	TRAT1,SH2D1A,FAIM3,ITK,CXCR2,CD19,LBP	9.14458E-07	6.63635E-05
GO_BP	GO:0006508	proteolysis	22	TLL1,CTSG,HPR,SFRP1,XPNPEP2,GZMK,ADAMTS8,CAPN6,ADAMDEC1,CTRL,RELN,CELA3B,CELA2B,CTRC,CPA2,MEP1A,PM20D1,CELA3A,CPA1,CPB1,CELA2A,CTRB2	1.09783E-06	6.97121E-05
GO_BP	GO:0007586	digestion	7	CTRL,AMY2B,MEP1A,PPY,AMY1A,CELA3A,CTRB2	1.93033E-06	0.000108957
GO_BP	GO:0007169	transmembrane receptor protein tyrosine kinase signaling pathway	9	FLT3,CD8A,CD8B,CD3E,ANGPTL1,ZAP70,ITK,BLK,STAP1	2.57525E-06	0.000130823
GO_BP	GO:0008343	adult feeding behavior	3	CARTPT,GHRL,LEP	4.4313E-06	0.000204646
GO_BP	GO:0002407	dendritic cell chemotaxis	4	CCL21,CCR7,CXCR2,CCL19	6.42641E-06	0.000272052
GO_BP	GO:1900004	negative regulation of serine-	2	CR1,SPINK1	6.98311E-06	0.000272878

		type endopeptidase activity				
GO_BP	GO:0019835	cytolysis	4	GZMH,GZMA,GZMB,C7	9.20132E-06	0.000333877
GO_BP	GO:0030217	T cell differentiation	5	CD3D,ZAP70,RHOH,PTPRC,BLK	1.90223E-05	0.000644221
GO_BP	GO:0001523	retinoid metabolic process	6	RBP2,APOA4,ADH4,APOA1,APOB,PNLIP	2.15444E-05	0.000684033
GO_BP	GO:0090023	positive regulation of neutrophil chemotaxis	4	CCL21,CCR7,CCL19,LBP	3.0666E-05	0.000916373
GO_BP	GO:0002548	monocyte chemotaxis	5	CCL14,CCL21,CCL19,CCL23,CCL15	3.38088E-05	0.000951064
GO_BP	GO:0002687	positive regulation of leukocyte migration	3	SELP,SELE,MADCAM1	3.93157E-05	0.000951064
GO_BP	GO:0006069	ethanol oxidation	3	ADH1B,ADH1A,ADH4	3.93157E-05	0.000951064
GO_BP	GO:0071276	cellular response to cadmium ion	3	MT1A,MT1H,MT1G	3.93157E-05	0.000951064
GO_BP	GO:0019915	lipid storage	4	CD36,FITM1,APOA1,CIDEA	5.04744E-05	0.001114825
GO_BP	GO:0030049	muscle filament sliding	4	DES,MYBPC2,MYBPC1,NEB	5.04744E-05	0.001114825
GO_BP	GO:2000147	positive regulation of cell motility	3	CCL21,CCR7,CCL19	5.8085E-05	0.001229465
GO_BP	GO:0001954	positive regulation	4	CD36,SFRP1,CCL21,CCR7	6.34893E-05	0.001290103

		of cell-matrix adhesion				
GO_BP	GO:0006959	humoral immune response	5	SH2D1A,PDCD1,POU2AF1,MS4A1,PAX5	7.7759E-05	0.001447576
GO_BP	GO:0007159	leukocyte cell-cell adhesion	4	SELP,SELE,CD40LG,APOA4	7.8947E-05	0.001447576
GO_BP	GO:0010744	positive regulation of macrophage derived foam cell differentiation	3	CD36,PLA2G2A,APOB	8.26372E-05	0.001447576
GO_BP	GO:0030193	regulation of blood coagulation	3	SCARA5,STAB2,GP1BA	8.26372E-05	0.001447576
GO_BP	GO:0007267	cell-cell signaling	11	SH2D1A,VIPR2,CARTPT,PCDH8,CCL21,LTB,CXCL13,CCL23,PPY,BHLHA15,CCL15	0.000102704	0.001690573
GO_BP	GO:0071347	cellular response to interleukin-1	6	CCL14,SFRP1,CCL21,CCL19,CCL23,CCL15	0.000103165	0.001690573
GO_BP	GO:0006935	chemotaxis	8	CXCR6,CCR7,CCR4,CXCR2,CXCR5,CCL23,FPR2,CCL15	0.000109447	0.00173747
GO_BP	GO:0017144	drug metabolic process	4	PCK1,ADH1A,CYP4F2,CYP3A4	0.000118403	0.001822693
GO_BP	GO:0008203	cholesterol metabolic process	6	APOA4,APOA1,CELA3B,APOB,LEP,CELA3A	0.000128845	0.001925091
GO_BP	GO:0071346	cellular response to interferon-gamma	5	CCL14,CCL21,CCL19,CCL23,CCL15	0.000138563	0.002011137

GO_ BP	GO:007135 6	cellular response to tumor necrosis factor	7	CCL14,SFRP1,CCL21,CCL19,CCL23,CCL15,A POB	0.000145 424	0.00205209
GO_ BP	GO:007129 4	cellular response to zinc ion	3	MT1A,MT1H,MT1G	0.000153 051	0.00210134 9
GO_ BP	GO:003018 3	B cell differentiati on	5	FLT3,GPR97,CD40LG,PTPRC,CR2	0.000180 57	0.00241393 8
GO_ BP	GO:000760 3	phototransd uction, visible light	6	RBP2,APOA4,GUCA1C,APOA1,APOB,PNLIP	0.000195 766	0.00254997 9
GO_ BP	GO:003016 8	platelet activation	11	SELP,P2RX1,PIK3R5,CD36,FIGF,PRKCB,CD40 LG,GP1BA,RASGRP2,IGF1,APOA1	0.000217 278	0.00275943 5
GO_ BP	GO:000758 4	response to nutrient	6	CD3E,CNR1,LCT,ADIPOQ,APOA1,HMGCS2	0.000238 439	0.00295432 1
GO_ BP	GO:003030 1	cholesterol transport	3	CD36,APOA1,APOB	0.000258 899	0.00313144 7
GO_ BP	GO:000600 6	glucose metabolic process	8	PCK1,PDK4,GHRL,PGM5,ADIPOQ,FBP2,AL DOB,LEP	0.000302 735	0.0035765
GO_ BP	GO:007134 5	cellular response to cytokine stimulus	3	FLT3,CCR7,CSF3	0.000327 874	0.00378544 9
GO_ BP	GO:003101 7	exocrine pancreas developmen t	2	PTF1A,IGF1	0.000364 052	0.00410974
GO_ BP	GO:000695 3	acute-phase response	4	REG3G,ITIH4,LBP,REG3A	0.000382 826	0.00422773
GO_ BP	GO:000715 5	cell adhesion	17	SELP,SELE,CHL1,MADCAM1,CD36,CNTN2, COL19A1,CD96,STAB2,PGM5,GP1BA,MYBPC 2,RELN,FPR2,SIGLEC14,NPHS1,MYBPC1	0.000401 794	0.00434279 9
GO_ BP	GO:001406 8	positive regulation of	5	SELP,FLT3,IGF1,RELN,CSF3	0.000458 137	0.00484861 8

		phosphatidylinositol 3-kinase signaling				
GO_BP	GO:0006956	complement activation	3	CFP,C7,FCN1	0.000503679	0.00522181
GO_BP	GO:0006957	complement activation, alternative pathway	2	CFP,C7	0.00053834	0.005362292
GO_BP	GO:0007494	midgut development	2	OTC,HMGCS2	0.00053834	0.005362292
GO_BP	GO:0008284	positive regulation of cell proliferation	16	FLT3,CCL14,FIGF,SFRP1,IL24,SLAMF1,VIP,CXCR2,MYOCD,IGF1,CNTFR,CSF3,FABP1,KLB,LEP,REG1A	0.000600491	0.005866334
GO_BP	GO:0007596	blood coagulation	17	SELP,P2RX1,SELE,PIK3R5,CD48,CD2,CD36,FIGF,PRKCB,GP1BA,RASGRP2,IGF1,SLC7A9,CYP4F2,APOA1,APOB,GATA5	0.000712222	0.006419181
GO_BP	GO:0030449	regulation of complement activation	3	CFP,CR1,C7	0.000737845	0.006419181
GO_BP	GO:0001771	immunological synapse formation	2	CCL21,CCL19	0.000758171	0.006419181
GO_BP	GO:0001878	response to yeast	2	VIP,PTX3	0.000758171	0.006419181
GO_BP	GO:0010001	glial cell differentiation	2	IGF1,RELN	0.000758171	0.006419181
GO_BP	GO:0010804	negative regulation of tumor necrosis factor-mediated	2	ADIPOQ,APOA1	0.000758171	0.006419181

		signaling pathway				
GO_ BP	GO:003010 1	natural killer cell activation	2	KLRK1,CD2	0.000758 171	0.00641918 1
GO_ BP	GO:200048 4	positive regulation of interleukin-8 secretion	2	CD2,FCN1	0.000758 171	0.00641918 1
GO_ BP	GO:003059 3	neutrophil chemotaxis	5	CCL14,CXCR2,CCL23,PLA2G1B,CCL15	0.000827 565	0.00689184 9
GO_ BP	GO:000716 5	signal transductio n	30	GFRA3,P2RX1,TRAT1,ZNF831,CHL1,KLRK1, MADCAM1,CD48,VIPR2,LYVE1,CARTPT,AS B2,SLAMF1,PRKCB,LTB,SIT1,PDCD1,ITK,CD 79B,CXCR2,RASGRP2,PENK,GUCA1C,IGF1,C NTFR,CCL23,RBPJL,PLA2G1B,CCL15,GABRP	0.000907 687	0.00743717 4
GO_ BP	GO:001923 3	sensory perception of pain	4	NGFR,CNR1,PENK,CNR2	0.000951 451	0.00767202
GO_ BP	GO:000695 8	complement activation, classical pathway	3	CR1,C7,CR2	0.001039 988	0.00821139 6
GO_ BP	GO:009879 2	xenophagy	6	PDK4,ASB2,HPR,FABP1,PPY,FCGR3B	0.001061 418	0.00821139 6
GO_ BP	GO:000715 7	heterophilic cell-cell adhesion via plasma membrane cell adhesion molecules	4	SELP,SELE,CADM3,REG3A	0.001066 835	0.00821139 6
GO_ BP	GO:003083 8	positive regulation of actin filament	4	CCL21,CCR7,CSF3,NPHS1	0.001192 277	0.00903995 2

		polymerization				
GO_BP	GO:0030212	hyaluronan metabolic process	3	LYVE1,STAB2,ITIH4	0.001219577	0.009110958
GO_BP	GO:0007218	neuropeptide signaling pathway	4	SORCS1,CARTPT,PENK,PPY	0.001328305	0.009779401
GO_BP	GO:0006000	fructose metabolic process	2	FBP2,ALDOB	0.001350961	0.009804115
GO_BP	GO:0016042	lipid catabolic process	5	PLA2G2A,PNLIPRP1,PLA2G2D,PLA2G1B,PNLIP	0.001393072	0.009878514
GO_BP	GO:0071222	cellular response to lipopolysaccharide	6	CD36,IL24,STAP1,CSF3,LBP,HMGCS2	0.001400104	0.009878514
GO_BP	GO:0006869	lipid transport	5	ABCA9,ABCA8,APOA4,APOA1,APOB	0.001510492	0.01051137
GO_BP	GO:0002227	innate immune response in mucosa	2	APOA4,PLA2G1B	0.001731458	0.01142312
GO_BP	GO:0010832	negative regulation of myotube differentiation	2	MYOCD,BHLHA15	0.001731458	0.01142312
GO_BP	GO:0090303	positive regulation of wound healing	2	REG3G,REG3A	0.001731458	0.01142312
GO_BP	GO:2000251	positive regulation of actin cytoskeleton	2	STAP1,CSF3	0.001731458	0.01142312

		reorganization				
GO_BP	GO:0006654	phosphatidic acid biosynthetic process	3	PLA2G2A,PLA2G2D,PLA2G1B	0.001886897	0.01228902
GO_BP	GO:0010466	negative regulation of peptidase activity	2	PI16,PI15	0.002172596	0.01397062
GO_BP	GO:0006629	lipid metabolic process	7	CD36,AOAH,PLIN1,PNLIPRP1,CEL,CYP3A4,CIDEA	0.002549757	0.01603127
GO_BP	GO:0002576	platelet degranulation	5	SELP,CD36,FIGF,IGF1,APOA1	0.002556168	0.01603127
GO_BP	GO:0007413	axonal fasciculation	2	CNTN2,CNR1	0.002677482	0.01619239
GO_BP	GO:0030214	hyaluronan catabolic process	2	LYVE1,STAB2	0.002677482	0.01619239
GO_BP	GO:0071398	cellular response to fatty acid	2	PDK4,HMGCS2	0.002677482	0.01619239
GO_BP	GO:0006874	cellular calcium ion homeostasis	5	CCL14,PRKCB,CCL19,CCL23,CCL15	0.002741203	0.01638272
GO_BP	GO:0002230	positive regulation of defense response to virus by host	6	PDK4,ASB2,HPR,FABP1,PPY,FCGR3B	0.002774866	0.01639107
GO_BP	GO:0030514	negative regulation of BMP signaling pathway	3	CHRDL2,CHRDL1,SFRP1	0.003120885	0.0182231

GO_BP	GO:0030889	negative regulation of B cell proliferation	2	BTLA,TNFRSF13B	0.003248978	0.01875546
GO_BP	GO:0008217	regulation of blood pressure	4	P2RX1,ATP1A2,CYP4F2,LEP	0.003390154	0.01927505
GO_BP	GO:0005975	carbohydrate metabolic process	13	PCK1,LYVE1,CHI3L2,STAB2,LCT,OGN,AMY2B,SI,FBP2,KLB,ALDOB,AMY2A,AMY1A	0.003497651	0.01927505
GO_BP	GO:0090630	activation of GTPase activity	3	CCL21,CCR7,CXCL13	0.003498549	0.01927505
GO_BP	GO:0006928	movement of cell or subcellular component	5	LYVE1,CXCR5,SERPINI2,IGF1,FPR2	0.003820714	0.02043077
GO_BP	GO:0007204	positive regulation of cytosolic calcium ion concentration	5	CD36,CCR7,CCR4,GHRL,CXCL13	0.003820714	0.02043077
GO_BP	GO:0010906	regulation of glucose metabolic process	2	PDK4,ADIPOQ	0.003889709	0.02058304
GO_BP	GO:0002526	acute inflammatory response	2	HPR,KLK1	0.004602076	0.02410159
GO_BP	GO:0006695	cholesterol biosynthetic process	3	APOA4,APOA1,HMGCS2	0.004815185	0.02470822
GO_BP	GO:0009267	cellular response to starvation	3	PDK4,CARTPT,SFRP1	0.004815185	0.02470822

GO_BP	GO:0002376	immune system process	2	CD300LG,FAIM3	0.005388263	0.02558166
GO_BP	GO:0007187	G-protein coupled receptor signaling pathway, coupled to cyclic nucleotide second messenger	2	CNR1,CNR2	0.005388263	0.02558166
GO_BP	GO:0007422	peripheral nervous system development	2	GFRA3,SOX10	0.005388263	0.02558166
GO_BP	GO:0009235	cobalamin metabolic process	2	CTRC,CTRB2	0.005388263	0.02558166
GO_BP	GO:0030534	adult behavior	2	SLC1A2,PAX5	0.005388263	0.02558166
GO_BP	GO:0071320	cellular response to cAMP	3	SLC26A3,ADIPOQ,AQP8	0.00585643	0.02754691
GO_BP	GO:0001654	eye development	2	CHRDL1,SLC39A5	0.007189812	0.03247736
GO_BP	GO:0006898	receptor-mediated endocytosis	6	CD36,SCARA5,HPR,STAB2,APOA1,APOB	0.007665442	0.03392789
GO_BP	GO:0006094	gluconeogenesis	3	PCK1,FBP2,ALDOB	0.007683224	0.03392789
GO_BP	GO:0006810	transport	11	P2RX1,RBP5,LYVE1,RBP2,PMP2,FABP4,ABCA8,LCN10,AQP8,FABP1,AQP12A	0.007747315	0.03392789
GO_BP	GO:0001816	cytokine production	2	ITK,FABP4	0.009307854	0.03865288

GO_ BP	GO:001010 7	potassium ion import	2	ATP1A2,KCNJ5	0.009307 854	0.03865288
GO_ BP	GO:007177 3	cellular response to BMP stimulus	2	SFRP1,GATA5	0.009307 854	0.03865288
GO_ BP	GO:001406 5	phosphatid ylinositol 3-kinase signaling	2	PIK3R5,IGF1	0.010488 98	0.04228889
GO_ BP	GO:001973 1	antibacterial humoral response	2	VIP,PLA2G1B	0.011752 99	0.04412902
GO_ BP	GO:190350 6	regulation of nucleic acid-templated transcriptio n	2	MYOCD,SLC26A3	0.011752 99	0.04412902
GO_ BP	GO:001635 8	dendrite developmen t	2	GHRL,RELN	0.013100 79	0.04533611
GO_ BP	GO:000961 1	response to wounding	3	NGFR,LYVE1,SLC1A2	0.013304 27	0.04533611
GO_ BP	GO:001046 8	regulation of gene expression	4	SCARA5,STAB2,RSPO1,IGF1	0.013767 48	0.04533611
GO_ BP	GO:190121 5	negative regulation of neuron death	2	CSF3,PM20D1	0.014533 16	0.04533611
GO_ BP	GO:001482 3	response to activity	2	PCK1,ADIPOQ	0.016050 72	0.04796332
GO_ BP	GO:007259 3	reactive oxygen species metabolic process	2	PDK4,AOX1	0.016050 72	0.04796332

GO_ BP	GO:200037 9	positive regulation of reactive oxygen species metabolic process	2	CD36,LEP	0.016050 72	0.04796332
GO_ BP	GO:000758 8	excretion	2	SLC26A3,NPHS1	0.019343 17	0.05282973
GO_ BP	GO:000166 6	response to hypoxia	6	FIGF,PRKCB,LCT,MYOCD,ADIPOQ,LEP	0.019697 51	0.05350981
GO_ BP	GO:000188 9	liver developmen t	4	ALDOB,LBP,OTC,HMGCS2	0.020633 34	0.05441126
GO_ BP	GO:003017 7	positive regulation of Wnt signaling pathway	2	SFRP1,RSPO1	0.021118 65	0.05441126
GO_ BP	GO:009877 9	mitophagy in response to mitochondri al depolarizati on	5	ASB2,HPR,FABP1,PPY,FCGR3B	0.021667 6	0.0550357
GO_ BP	GO:000716 2	negative regulation of cell adhesion	2	ADAMDEC1,ARHGDIG	0.024928 68	0.05917649
GO_ BP	GO:000176 4	neuron migration	4	GFRA3,CHL1,CCR4,RELN	0.025891 17	0.06117541
GO_ BP	GO:001648 5	protein processing	3	GZMH,CTSG,GZMB	0.026454 38	0.06221679
GO_ BP	GO:001004 3	response to zinc ion	2	ALDOB,OTC	0.026963 12	0.06312104
GO_ BP	GO:000725 7	activation of JUN kinase activity	2	MAP4K1,CCL19	0.029083 61	0.06379522

GO_ BP	GO:003015 4	cell differentiati on	10	FLT3,TLL1,CHRDL2,KLRK1,FAM65B,CHRDL 1,COL19A1,ZAP70,FIGF,FCRLA	0.029134 82	0.06379522
GO_ BP	GO:000224 4	hematopoie tic progenitor cell differentiati on	3	SFRP1,PTPRC,TNFRSF13B	0.031027 28	0.06764747
GO_ BP	GO:000681 1	ion transport	5	P2RX1,SLC1A2,SLC26A3,SLC7A9,GABRP	0.034496 17	0.0730169
GO_ BP	GO:000751 9	skeletal muscle tissue developmen t	2	COL19A1,NPHS1	0.040962 93	0.0831605
GO_ BP	GO:000751 7	muscle organ developmen t	3	FAM65B,IGF1,NEB	0.043402 46	0.08683866
GO_ BP	GO:000663 5	fatty acid beta- oxidation	2	ADIPOQ,LEP	0.043590 27	0.08683866
GO_ BP	GO:001972 2	calcium- mediated signaling	2	SELE,BHLHA15	0.043590 27	0.08683866
GO_ BP	GO:003112 4	mRNA 3- end processing	186	ACKR1,SELP,MPZ,FLT3,CCL14,PLP1,GFRA3, PCK1,CHRDL2,MYRIP,P2RX1,TRAT1,PDK4,S ELE,CD8A,GPR171,CHL1,GZMA,CD247,SOX 10,KLRK1,PIK3R5,MADCAM1,CD48,SH2D1A ,CD8B,BTLA,CD3D,CTSG,CD2,GZMB,CFP,N GFR,VIPR2,CD36,LYVE1,CNTN2,CXCR6,CD3 E,CD300LG,NLRP6,CARTPT,SEPT1,ASB2,KL RB1,SCARA5,CD96,FITM1,ADH1B,P2RY14,Z AP70,THEMIS,FIGF,RHOH,SFRP1,IL24,CAD M3,SLAMF1,SLAMF6,CCL21,AOAH,NCF1B, CCR7,FAIM3,PRKCB,ABCA9,CCR4,VIP,ADH 1A,HLA- DQA1,LTB,GHRL,STAB2,LY9,CNR1,PMP2,IC OS,SLC1A2,PDCD1,CLEC10A,CD40LG,TLR10	0.043991 57	0.08729577

				,ITK,MAP4K1,AOX1,LCT,SLIT3,P2RY10,NCF1C,CD79B,PLIN1,FABP4,IRF4,CXCR2,ZBTB16,CCL19,GNMT,MEDAG,CR1,CXCL13,GP1BA,RSPO1,PTPRC,ADAMTS8,PTF1A,CD3G,RASGRP2,MYOCD,CXCR5,ABCA8,CAPN6,LMO3,MT1A,CUZD1,CLEC4E,SLC26A3,APOA4,BLK,MT1H,STMN4,ADH4,GUCA1C,IGF1,TNFRSF13B,CNTFR,RELN,C16orf89,OGN,SLC7A9,CCL23,PLA2G2A,MT1G,MS4A1,ATP1A2,CYP4F2,STAP1,CNR2,ADIPOQ,AQP8,ERP27,FCER2,CD19,APOA1,CSF3,SI,KCNJ5,ARHGDIG,SLC39A5,REG3G,SIGLEC14,FBP2,PAX5,ITIH4,CTRC,C7,FABP1,CEL,KLB,PTX3,ALDOB,RBPJL,LBP,OTC,AMY2A,PLA2G2D,PRPH,CYP3A4,GSTA2,CR2,BHLHA15,PLA2G1B,CCL15,GABRP,SPINK1,NPHS1,APOB,LEP,SYCE1,CIDEA,FCN1,HMGCS2,CTRB2,GATA5,PDIA2,PNLIP,KLK1						
GO_BP	GO:0007229	integrin-mediated signaling pathway	3	PLP1,MADCAM1,APOA1	0.0494408	0.09589929				
GO_BP	GO:0006805	xenobiotic metabolic process	6	ADH1B,ADH1A,ADH4,CYP4F2,CYP3A4,GSTA2	0.02038456	0.05441126	3	UGT1A9,CYP2W1,CYP26A1	0.002242658	0.009184218
GO_BP	GO:0008344	adult locomotory behavior	2	CHL1,ATP1A2	0.0462998	0.09046268	2	NTSR1,NTF4	0.000563753	0.006782863
GO_BP	GO:0008283	cell proliferation					3	FAM83A,KRT16,GCG	0.03128408	0.04761824
GO_BP	GO:0030198	extracellular matrix organization					3	HAPLN1,TTR,COL17A1	0.02336775	0.03864666
GO_BP	GO:0007399	nervous system development					3	PCSK2,CPLX2,BARX1	0.009626541	0.01971149

GO_BP	GO:0007160	cell-matrix adhesion	2	MSLN,COL17A1	0.004457819	0.01245765
GO_BP	GO:0006919	activation of cysteine-type endopeptidase activity involved in apoptotic process	2	CDKN2A,CASP14	0.003637634	0.01097672
GO_BP	GO:0007268	synaptic transmission	4	GRM4,CRH,KCNK16,NTSR1	0.003403038	0.0108168
GO_BP	GO:0006493	protein O-linked glycosylation	2	MUC21,GALNT5	0.002698657	0.01054929
GO_BP	GO:0030855	epithelial cell differentiation	2	UPK2,BARX1	0.002014519	0.008884546
GO_BP	GO:0016266	O-glycan processing	2	MUC21,GALNT5	0.001379223	0.007413321
GO_BP	GO:0007269	neurotransmitter secretion	2	GRM4,WNT7A	0.000785472	0.006782863
GO_BP	GO:0030819	positive regulation of cAMP biosynthetic process	2	CRH,GCG	6.91768E-05	0.001699772
GO_BP	GO:0007612	learning	3	GRM4,TH,NTSR1	3.72165E-05	0.001066874
GO_BP	GO:0090280	positive regulation of calcium ion import	2	CRH,GCG	1.17287E-05	0.000403468
GO_BP	GO:0001963	synaptic transmissio	2	CRH,TH	1.89172E-06	8.13439E-05

		n, dopaminerg ic					
GO_ BP	GO:001814 9	peptide cross-linking		3	SPRR2A,SPRR1B,SPRR3	9.24892E -07	5.30271E -05
GO_ BP	GO:003021 6	keratinocyte differentiati on		4	SPRR2A,KRT16,SPRR1B,SPRR3	6.94754E -07	5.30271E -05
GO_ BP	GO:000854 4	epidermis developmen t		8	KLK5,SPRR2A,KRT16,SPRR1B,NTF4,SPRR3,COL1 7A1,CASP14	1.69702E -12	2.91887E -10

Table S4. Enrichment for cell types and or oncogenic pathway activities in "head" vs. "body/tail" samples. Using the 20 Gatza's activation pathway signature [34] and the 64 immunes and stroma cell types classifiers using the xCell web tool [35].

			PAAD Location				
**Base**	**GES**	**N**	**body/tail**	**head**	**p-value**	**Statistic**	**Enrichement**
xCell	CD8pos_Tcm	249	0.01 (0-0.12)	0.04 (0-0.2)	0.000	-4.2	Enriched in "Head"
xCell	naive_B.cells	249	0.01 (0-0.06)	0.02 (0-0.26)	0.000	-4	Enriched in "Head"
xCell	pDC	249	0.01 (0-0.07)	0.02 (0-0.23)	0.000	-4	Enriched in "Head"
xCell	Platelets	249	0.03 (0-0.1)	0.05 (0-0.13)	0.000	-4	Enriched in "Head"
xCell	B.cells	249	0.07 (0-0.24)	0.12 (0-0.65)	0.000	-3.7	Enriched in "Head"
xCell	CD4pos_T.cells	249	0.07 (0-0.24)	0.1 (0-0.42)	0.000	-3.7	Enriched in "Head"
xCell	HSC	249	0.1 (0-0.38)	0.16 (0-0.46)	0.000	-3.7	Enriched in "Head"
xCell	Microenvironment_Score	249	0.69 (0.37-1.22)	0.82 (0.3-1.66)	0.000	-3.7	Enriched in "Head"
xCell	Immune_Score	249	0.51 (0.23-0.95)	0.61 (0.21-1.38)	0.001	-3.6	Enriched in "Head"
xCell	Keratinocytes	249	0.35 (0.11-0.51)	0.3 (0.04-0.49)	0.001	3.6	Enriched in "B/T"
xCell	Adipocytes	249	0.06 (0-0.21)	0.09 (0-0.41)	0.001	-3.5	Enriched in "Head"
Gatza	E2F1	249	0.31 (0-0.9)	0.46 (0-0.99)	0.001	-3.4	Enriched in "Head"
xCell	Memory_B.cells	249	0.01 (0-0.15)	0.03 (0-0.3)	0.001	-3.3	Enriched in "Head"
xCell	Epithelial_cells	249	0.69 (0.39-0.79)	0.64 (0.2-0.79)	0.002	3.3	Enriched in "B/T"
xCell	Preadipocytes	249	0.03 (0-0.13)	0.05 (0-0.24)	0.002	-3.2	Enriched in "Head"
xCell	CD4pos_naive_T.cells	249	0.22 (0.07-0.39)	0.25 (0.07-0.66)	0.004	-3	Enriched in "Head"
xCell	aDC	249	0.19 (0.06-0.3)	0.22 (0.06-0.32)	0.005	-2.9	Enriched in "Head"
xCell	CD8pos_T.cells	249	0.04 (0-0.21)	0.06 (0-0.24)	0.005	-2.9	Enriched in "Head"
xCell	Macrophages_M2	249	0.17 (0-0.38)	0.21 (0.02-0.46)	0.009	-2.7	Enriched in "Head"
xCell	Megakaryocytes	249	0.23 (0.14-0.34)	0.25 (0.09-0.38)	0.012	-2.6	Enriched in "Head"
xCell	Smooth_muscle	249	0.44 (0.32-0.6)	0.41 (0.22-0.63)	0.012	2.6	Enriched in "B/T"
xCell	Tregs	249	0.02 (0-0.11)	0.03 (0-0.14)	0.027	-2.3	Enriched in "Head"
Gatza	TGFB	249	0.83 (0.52-0.93)	0.8 (0.5-0.93)	0.030	2.2	Enriched in "B/T"
xCell	CD4pos_memory_T.cells	249	0.23 (0.13-0.38)	0.25 (0.09-0.54)	0.030	-2.2	Enriched in "Head"
xCell	CD4pos_Tem	249	0.01 (0-0.09)	0.02 (0-0.2)	0.036	-2.1	Enriched in "Head"
xCell	Class.switched_memory_B.cells	249	0.15 (0-0.27)	0.18 (0-0.58)	0.039	-2.1	Enriched in "Head"
Gatza	IFNg	249	0.6 (0.18-0.99)	0.68 (0.08-1)	0.039	-2.1	Enriched in "Head"
xCell	StromaScore	249	0.18 (0.05-0.3)	0.2 (0.04-0.5)	0.044	-2	Enriched in "Head"
Gatza	Acidosis	249	0.36 (0.01-0.87)	0.45 (0.01-0.97)	0.045	-2	Enriched in "Head"
xCell	Neutrophils	249	0.03 (0-0.15)	0.05 (0-0.2)	0.047	-2	Enriched in "Head"
xCell	Macrophages	249	0.13 (0-0.26)	0.15 (0-0.4)	0.051	-2	
xCell	Macrophages_M1	249	0.02 (0-0.06)	0.02 (0-0.1)	0.058	-1.9	
xCell	Astrocytes	249	0.01 (0-0.06)	0 (0-0.08)	0.059	1.9	

xCell	Fibroblasts	249	0.07 (0-0.28)	0.09 (0-0.43)	0.072	-1.8
Gatza	PI3K	249	0.62 (0.07-0.98)	0.7 (0.01-1)	0.080	-1.8
Gatza	Hypoxia	249	0.36 (0.02-0.97)	0.28 (0.01-0.97)	0.081	1.8
Gatza	GlucoseDepriv	249	0.5 (0.03-1)	0.59 (0-1)	0.091	-1.7
xCell	DC	249	0.11 (0-0.22)	0.13 (0-0.29)	0.110	-1.6
xCell	Erythrocytes	249	0 (0-0.06)	0 (0-0.04)	0.112	1.6
xCell	Osteoblast	249	0.01 (0-0.11)	0 (0-0.14)	0.127	1.6
Gatza	RAS	249	0.24 (0.06-0.57)	0.21 (0.01-0.71)	0.135	1.5
Gatza	P63	249	0.48 (0-0.96)	0.41 (0-1)	0.142	1.5
Gatza	AKT	249	0.44 (0-0.98)	0.52 (0-1)	0.149	-1.5
xCell	CD8pos_Tem	249	0.01 (0-0.16)	0.02 (0-0.21)	0.162	-1.4
xCell	Th2_cells	249	0.03 (0-0.12)	0.04 (0-0.12)	0.165	-1.4
Gatza	EGFR	249	0.84 (0.48-0.99)	0.81 (0.14-0.98)	0.197	1.3
xCell	Pericytes	249	0.18 (0.08-0.25)	0.18 (0.08-0.27)	0.201	1.3
Gatza	PR	249	0.33 (0.06-0.94)	0.27 (0.01-0.84)	0.205	1.3
xCell	MPP	249	0.19 (0-0.67)	0.22 (0-0.94)	0.207	-1.3
xCell	Skeletal_muscle	249	0.02 (0-0.04)	0.02 (0-0.07)	0.212	-1.3
xCell	NK_cells	249	0.01 (0-0.08)	0 (0-0.07)	0.221	1.2
xCell	Sebocytes	249	0.03 (0.02-0.05)	0.03 (0.01-0.05)	0.241	1.2
Gatza	MYC	249	0.28 (0.01-0.92)	0.23 (0.02-0.91)	0.242	1.2
Gatza	IFNa	249	0.52 (0.02-0.99)	0.58 (0.01-0.99)	0.259	-1.1
xCell	cDC	249	0.06 (0-0.24)	0.08 (0-0.29)	0.267	-1.1
xCell	Melanocytes	249	0.06 (0.01-0.1)	0.06 (0-0.13)	0.293	-1.1
Gatza	TNFa	249	0.51 (0.04-0.98)	0.46 (0-0.99)	0.304	1
xCell	MSC	249	0.51 (0-0.86)	0.55 (0-1.15)	0.311	-1
xCell	ly_Endothelial_cells	249	0.16 (0.08-0.32)	0.17 (0-0.33)	0.318	-1
xCell	CD4pos_Tcm	249	0.04 (0-0.12)	0.03 (0-0.23)	0.337	0.97
xCell	pro_B.cells	249	0.02 (0-0.13)	0.03 (0-0.16)	0.378	-0.89
xCell	Mast_cells	249	0.07 (0.04-0.1)	0.07 (0.03-0.12)	0.409	0.83
Gatza	STAT3	249	0.66 (0.01-1)	0.63 (0.04-0.99)	0.415	0.82
Gatza	LacticAcidosis	249	0.82 (0.2-0.98)	0.8 (0.44-0.99)	0.427	0.8
xCell	Hepatocytes	249	0.08 (0-0.16)	0.08 (0-0.23)	0.452	-0.76
xCell	Plasma_cells	249	0.01 (0-0.07)	0.01 (0-0.1)	0.457	0.75
xCell	Endothelial_cells	249	0.22 (0.11-0.38)	0.22 (0.04-0.37)	0.475	0.72
xCell	GMP	249	0 (0-0.08)	0 (0-0.07)	0.589	0.54
xCell	CD8pos_naive_T.cells	249	0.14 (0.07-0.24)	0.14 (0.04-0.3)	0.629	0.49
xCell	Chondrocytes	249	0.3 (0-0.42)	0.3 (0.04-0.53)	0.675	-0.42
xCell	Basophils	249	0.06 (0-0.27)	0.06 (0-0.24)	0.681	0.41
xCell	CMP	249	0 (0-0.05)	0.01 (0-0.08)	0.699	-0.39
Gatza	P53	249	0.53 (0.04-0.99)	0.55 (0.07-0.98)	0.709	-0.38

xCell	Monocytes	249	0.06 (0-0.24)	0.06 (0-0.27)	0.730	-0.35
xCell	Myocytes	249	0.01 (0-0.06)	0 (0-0.06)	0.737	0.34
xCell	NKT	249	0.2 (0-0.37)	0.21 (0-0.44)	0.752	-0.32
Gatza	SRC	249	0.28 (0.01-0.87)	0.26 (0-0.92)	0.762	0.3
xCell	Eosinophils	249	0.17 (0-0.38)	0.18 (0-0.44)	0.788	-0.27
xCell	Mesangial_cells	249	0.02 (0-0.07)	0.02 (0-0.07)	0.821	0.23
Gatza	ER	249	0.36 (0-0.95)	0.35 (0-1)	0.847	0.19
Gatza	BCAT	249	0.25 (0.01-0.83)	0.24 (0.01-0.87)	0.850	0.19
xCell	MEP	249	0 (0-0.03)	0 (0-0.09)	0.904	0.12
xCell	CLP	249	0.52 (0.45-0.66)	0.52 (0.39-0.66)	0.909	-0.11
xCell	mv_Endothelial_cells	249	0.28 (0.19-0.45)	0.28 (0.1-0.45)	0.923	0.097
xCell	Neurons	249	0.04 (0-0.1)	0.04 (0-0.16)	0.926	0.093
xCell	iDC	249	0.08 (0-0.18)	0.08 (0-0.21)	0.927	-0.093
Gatza	HER2	249	0.75 (0.05-0.97)	0.75 (0.12-0.98)	0.952	-0.06
